# Physician, Practice, and Patient Characteristics Associated With Biosimilar Use in Medicare Recipients

**DOI:** 10.1001/jamanetworkopen.2020.34776

**Published:** 2021-01-27

**Authors:** Emma Boswell Dean, Phyllis Johnson, Amelia M. Bond

**Affiliations:** 1 Department of Health Management and Policy, Miami Business School, University of Miami, Miami, Florida; 2 Division of Health Policy and Economics, Department of Population Health Sciences, Weill Cornell Medical College, New York, New York

## Abstract

**Importance:**

Biosimilars, or highly similar versions of complex biologic drugs, have the potential to slow drug spending growth; however, biosimilar uptake in the United States has been slow. Little is known about barriers to biosimilar uptake following drug launch.

**Objective:**

To examine the patient, physician, and practice characteristics associated with biosimilar use in the Medicare population.

**Design, Setting, and Participants:**

This cross-sectional study used regression analysis to estimate the association between biosimilar use and various characteristics. Medicare fee-for-service beneficiaries who received a filgrastim product or an infliximab product between the launch of a class’s first biosimilar (quarter 3 2015 for filgrastim-sndz and quarter 4 2016 for infliximab-dyyb) and December 2018. Data analysis was conducted from March to November 2020.

**Exposures:**

Patient demographic characteristics and product clinical indications; physician demographic characteristics, specialty, and volume of filgrastim or infliximab biologic administration; hospital size, ownership, 340B status, academic medical center status, and system affiliation; physician office size and multispecialty status.

**Main Outcomes and Measures:**

Administration of a filgrastim or infliximab biosimilar.

**Results:**

The final filgrastim sample included 25 870 patients (11 857 [45.8%] men; 14 224 [55.0%] aged 65-74 years; 22 617 [87.4%] White individuals) who had 259 178 administrations (79 017 [30.5%] biosimilar administrations), and the final infliximab sample included 14 786 patients (4765 [32.2%] men; 8773 [59.3%] aged 65-74 years; 13 467 [91.1%] White individuals) who had 174 973 administrations (9012 [5.2%] biosimilar administrations). In adjusted analyses, no patient demographic characteristics and 2 of 9 clinical indications (22.2%) were associated with biosimilar use (filgrastim, neutropenia: adjusted difference, −2.0 [95% CI, −3.9 to −0.2] percentage points; *P* = .03; infliximab, Crohn disease: adjusted difference, −1.8 [95% CI, −2.9 to −0.8] percentage points; *P* = .001). Several physician characteristics were associated with biosimilar administrations, including high filgrastim or infliximab prescribing volume (high vs low volume, filgrastim: adjusted difference, 3.6 [95% CI, 1.5 to 5.8] percentage points; *P* = .001; infliximab: adjusted difference, 1.2 [95% CI, 0.3 to 2.2] percentage points; *P* = .007) and specialty (eg, hematologist-oncologists vs primary care, filgrastim: adjusted difference, −3.0 [95% CI, −5.4 to −0.5] percentage points; *P* = .02). Numerous practice characteristics were associated with biosimilar use, including practice setting (outpatient hospital department vs office practice, filgrastim: adjusted difference, −16.1 [95% CI, −18.1 to −14.1] percentage points; *P* < .001; infliximab: adjusted difference, 3.0 [95% CI, 2.2 to 3.7] percentage points; *P* < .001) and hospital outpatient department ownership status (for-profit vs not-for-profit, filgrastim: adjusted difference, −17.4 [95% CI, −21.6 to −13.3] percentage points; *P* < .001; infliximab: adjusted difference, 10.8 [95% CI, 6.7 to 14.9] percentage points; *P* < .001).

**Conclusions and Relevance:**

In this study, practice setting and hospital ownership status had the largest associations with biosimilar usage, suggesting practices play a role in steering physicians toward certain medications. However, the types of practices with high biosimilar use differed by drug class. Further research is needed to understand the reasons for these differences across drug classes.

## Key Points

**Question:**

What patient, physician, and practice characteristics are associated with biosimilar usage for the biologics filgrastim and infliximab?

**Findings:**

In this cross-sectional study of 40 656 Medicare fee-for-service beneficiaries, few patient and physician characteristics were associated with biosimilar usage. Practice setting characteristics had the largest associations; however, the types of practices with high biosimilar use differed by drug class.

**Meaning:**

In this study, practice setting and hospital ownership status were associated with use of biosimilars, but further research is needed to understand the reasons for differences across drug classes.

## Teaser

This cross-sectional study examines patient, physician, and practice characteristics associated with biosimilar usage in the Medicare population.

## Introduction

Biologic medicines—complex medicines derived from biologic sources—account for a growing share of drug spending.^1^ Biologic medicine spending in the United States increased by 50.1%^2^ between 2014 and 2018, putting additional financial pressure on public and private payers. In 2010, the Biologics Price Competition and Innovation Act created a pathway for the development, approval, and launch of highly similar biosimilar medications to compete with originator biologic medications.^3^ Similar to the role of generic medicines for small-molecule drugs, biosimilars are intended as a substitute for biologic drugs, thus creating competition in the biologics market to drive spending down.

Biosimilars have the potential to decrease drug spending growth; however, products have been slow to launch in the United States. As of June 30, 2020, 27 biosimilars have been approved and 14 have launched, compared with 58 approvals in Europe.^4,5^ Even when launched, biosimilar uptake in the United States has been slow.^6,7,8,9^ Identifying characteristics associated with biosimilar use can clarify which barriers are likely driving the slow adoption^10,11,12,13,14^ and may help to develop policies to encourage the uptake of biosimilars.

This study asks what patient, physician, and practice characteristics are associated with biosimilar usage for the biologics filgrastim and infliximab in the Medicare fee-for-service population through 2018. Two previous studies examined characteristics associated with filgrastim biosimilar uptake in the Medicare fee-for-service population through 2017^15^ and in the commercial and Medicare Advantage settings through 2018.^9^ Two studies^6,16^ have examined uptake of infliximab through 2018 in the Medicare fee-for-service population. This study extends previous analysis for the filgrastim drug class in the Medicare fee-for-service population and is the first to examine associations with practice characteristics and a wider range of physician characteristics in the infliximab drug class.

## Methods

### Data and Sample

The primary data source was claims and enrollment data from 2014 to 2018 for a random 20% sample of beneficiaries enrolled in Parts A and B of fee-for-service Medicare. All Part B biosimilar administrations were identified for filgrastim products (filgrastim, filgrastim-sndz, and close competitor tbo-filgrastim) and for infliximab products (infliximab, infliximab-dyyb, and infliximab-abda). Primary analysis focused on administrations beginning in the quarter of biosimilar launches in each drug class through the end of 2018 (quarter 3 2015 to quarter 4 2018 for filgrastim drug class and quarter 4 2016 to quarter 4 2018 for infliximab drug class).

This study was deemed exempt from review by the institutional review board at Weill Cornell Medical College, including a waiver of informed consent due to the use of secondary data. This study follows the Strengthening the Reporting of Observational Studies in Epidemiology (STROBE) reporting guideline for cross-sectional studies.^17^

### Outcomes and Covariates

The primary outcome was a binary variable indicating whether a Part B biologic administration was a biosimilar. Patient characteristics included indicators for age group (ie, 65-74 or ≥75 years), sex, race, dual Medicare-Medicaid eligibility, relevant medical conditions, and risk scores. Risk scores were based on the Health and Human Services Hierarchical Conditions Categories risk adjustment model using claims from the preceding year.^14^ For patients receiving biosimilars, we identified whether a patient had previously used the originator biologic. Characteristics were present at the time of a patient’s first administration (biosimilar or originator biologic) in the sample.

The administering physician was identified on a beneficiary’s biologic claim (eAppendix in the Supplement). Physician characteristics included age, sex, and indicators for physician specialty from the 2018 Physician Compare database. Additionally, indicators for whether a physician practiced in a hospital-owned practice and low, medium, or high biologic administration volume were constructed from Medicare claims (Table 1). If a physician only appeared in years prior to 2018, physician characteristics were collected from the relevant year of Physician Compare data. Finally, a physician’s percentage of biosimilar prescriptions during the full sample period was calculated for physicians who administered any biosimilars.

**Table 1.** Characteristics of Physicians Who Administer Biologics by Biosimilar Administration, Filgrastim and Infliximab Drug Classes^a^

Characteristic	Filgrastim sample (Q3 2015-Q4 2018)				Infliximab sample (Q4 2016-Q3 2018)			
	No. (%)		Absolute difference, % (95% CI)^d^	*P* value	No. (%)		Absolute difference, % (95% CI)^d^	*P* value
	≥1 (n = 5713)^b^	0 (n = 9258)^c^			≥1 (n = 1106)^b^	0 (n = 9212)^c^		
Biosimilar administration rate, mean (SD)	76.6 (31.6)	NA	NA	NA	49.4 (34.4)	NA	NA	NA
Years in practice^e^								
<15	1078 (18.8)	1800 (19.4)	−0.5 (−1.9 to 0.7)	.40	196 (17.7)	1653 (17.9)	−0.2 (−3.2 to 1.5)	.88
15-30	2711 (47.4)	4229 (45.7)	1.8 (−0.1 to 3.4)	.04	468 (42.3)	3957 (43.0)	−0.7 (−3.8 to 2.4)	.65
>30	1924 (33.7)	3229 (34.9)	−1.2 (−2.7 to 0.4)	.14	442 (40.0)	3602 (39.1)	0.9 (−2.2 to 4.0)	.56
Sex								
Men	4027 (70.5)	6512 (70.3)	0.1 (−1.4 to 1.7)	.85	801 (72.4)	6367 (69.1)	3.3 (0.5 to 6.1)	.02
Women	1686 (29.5)	2746 (29.7)	−0.1 (−1.7 to 1.4)	.85	305 (27.6)	2845 (30.9)	−3.3 (−6.1 to −0.5)	.02
Practicing in hospital-owned practice^f^	716 (12.5)	1299 (14.0)	−1.5 (−2.6 to −0.4)	.009	453 (41.0)	2679 (29.1)	11.9 (8.8 to 14.9)	<.001
Specialty								
Filgrastim-related								
Hematologist- oncologist	1882 (32.9)	2577 (27.8)	5.1 (3.6 to 6.6)	<.001	NA	NA	NA	NA
Oncologist	869 (15.2)	1419 (15.3)	−0.1 (−1.3 to 1.1)	.85	NA	NA	NA	NA
Other cancer related^g^	528 (9.2)	782 (8.4)	0.8 (−0.1 to 1.7)	.09	NA	NA	NA	NA
Primary care	1516 (26.5)	2619 (28.3)	−1.8 (−3.2 to −0.3)	.02	NA	NA	NA	NA
Other	918 (16.1)	1861 (20.1)	−4.0 (−5.3 to −2.8)	<.001	NA	NA	NA	NA
Infliximab-related								
Dermatologist	NA	NA	NA	NA	14 (1.3)	108 (1.2)	0.1 (−0.6 to 0.8)	.79
Gastroenterologist	NA	NA	NA	NA	190 (17.2)	1775 (19.3)	−2.1 (−4.5 to 0.3)	.10
Rheumatologist	NA	NA	NA	NA	414 (37.4)	2193 (23.8)	13.6 (10.6 to 16.6)	<.001
Primary care	NA	NA	NA	NA	320 (28.9)	3867 (42.0)	−13.0 (−15.9 to −10.2)	<.001
Other	NA	NA	NA	NA	168 (15.2)	1269 (13.8)	1.4 (−0.8 to 3.6)	.20
Volume of biologic^h^								
Low-volume prescriber	1012 (17.9)	2804 (30.3)	−12.4 (−13.8 to −11.0)	<.001	414 (37.4)	5081 (55.2)	−17.7 (−20.8 to −14.6)	<.001
Medium-volume prescriber	3306 (57.7)	4728 (51.1)	6.6 (5.0 to 8.2)	<.001	340 (30.7)	2516 (27.3)	3.4 (0.6 to 6.2)	.02
High-volume prescriber	1395 (24.5)	1726 (18.6)	5.8 (4.5 to 7.1)	<.001	352 (31.8)	1616 (17.5)	14.3 (11.9 to 16.8)	<.001

Abbreviations: NA, not applicable; Q, quarter.

^a^ Samples include all physicians who administered a biologic since biosimilar launch in the drug class (Q3 2015 for filgrastim and Q4 2016 for infliximab).

^b^ Physician administered at least 1 biosimilar in the drug class during the sample period.

^c^ Physician never administered a biosimilar in the drug class during the sample period.

^d^ Comparisons reflect 2-group test of proportions.

^e^ Years in practice indicates years since completion of medical school.

^f^ A physician was categorized as practicing in a hospital-owned practice if at least 90% of their claims were billed in a hospital outpatient department.^18^

^g^ Other cancer-related specialties include gastroenterology, gynecology, proctology, urology, nephrology, endocrinology, and hematology.

^h^ For filgrastim sample, low, medium, and high volume were defined as at least 1, more than 1 to 5, and more than 5 average monthly filgrastim administrations, respectively. For infliximab, low, medium, and high volume were defined as at least 1, more than 1 to 2, and more than 2 average monthly infliximab administrations, respectively.

Biosimilars may be administered in the hospital outpatient department setting or a physician’s office. Practice characteristics were measured separately for each setting. In the hospital outpatient setting, characteristics included indicators for hospital size (<50, 50-100, and ≥100 beds), ownership (not-for-profit, for-profit, and government), academic medical center status and system affiliation from the 2018 American Hospital Association Survey, and 340B status in 2018 from the Health Resources and Services Administration Office of Pharmacy Affairs. In the office setting, characteristics included indicators for practice size (1-5, 6-19, 20-99, and ≥100 physicians) and multispecialty status (Table 2).^18^ The percentage of biosimilar prescriptions during the full sample was calculated for hospital outpatient departments or offices that administered at least 1 biosimilar medication.

**Table 2.** Characteristics of Practices That Administer Biologics by Biosimilar Administration, Filgrastim and Infliximab Drug Classes^a^

Characteristic	Filgrastim sample (Q3 2015-Q4 2018)				Infliximab sample (Q4 2016-Q4 2018)			
	≥1^b^	0^c^	Difference in means (95% CI)^d^	*P* value	≥1^b^	0^c^	Difference in means (95% CI)^d^	*P* value
**Office setting**								
No.	327	636	NA	NA	197	1175	NA	NA
Biosimilar administration rate, mean (SD)	50.9 (32.2)	NA	NA	NA	33.2 (30.6)	NA	NA	NA
Practice size, No. of physicians^e^								
≤5	150 (46.0)	366 (57.5)	−11.5 (−18.2 to −4.9)	.001	105 (53.6)	638 (54.3)	−0.7 (−8.3 to 6.8)	.85
6-19	74 (22.7)	79 (12.4)	10.3 (5.1 to 15.5)	<.001	33 (16.8)	196 (16.7)	0.2 (−5.5 to 5.8)	.96
20-99	59 (18.1)	81 (12.7)	5.4 (0.4 to 10.3)	.03	27 (13.8)	192 (16.3)	−2.6 (−7.8 to 2.7)	.36
≥100	43 (13.2)	110 (17.3)	−4.1 (−8.8 to 0.6)	.10	31 (15.8)	149 (12.7)	3.1 (−2.3 to 8.6)	.23
Multispecialty^f^	191 (58.6)	320 (50.3)	8.3 (1.7 to 14.9)	.02	84 (42.9)	468 (39.8)	3.0 (−4.4 to 10.5)	.42
**HOPD setting**								
No.	558	1525	NA	NA	362	1322	NA	NA
Biosimilar administration rate, mean (SD)	59.3 (31.5)	NA	NA	NA	37.4 (29.0)	NA	NA	NA
Beds, No.								
<50	76 (13.6)	425 (27.9)	−14.2 (−17.9 to −10.6)	<.001	43 (11.9)	317 (24.0)	−12.1 (−16.1 to −8.0)	<.001
51-100	50 (9.0)	199 (13.0)	−4.1 (−7.0 to −1.2)	.01	49 (13.6)	150 (11.3)	2.2 (−1.7 to 6.2)	.25
101-250	179 (32.1)	443 (29.0)	3.1 (−1.4 to 7.6)	.17	131 (36.3)	381 (28.8)	7.5 (1.9 to 13.0)	.006
>250	252 (45.2)	458 (30.0)	15.2 (10.5 to 19.9)	<.001	138 (38.2)	474 (35.9)	2.4 (−3.3 to 8.0)	.41
Ownership status								
Not-for-profit	461 (82.8)	1041 (68.3)	14.5 (10.6 to 18.4)	<.001	258 (71.5)	990 (74.9)	−3.4 (−8.6 to 1.8)	.19
For-profit	10 (1.8)	201 (13.2)	−11.4 (−13.4 to −9.4)	<.001	60 (16.6)	89 (6.7)	9.9 (−5.8 to 14.0)	<.001
Government	86 (15.4)	283 (18.6)	−3.1 (−6.7 to 0.5)	.10	43 (11.9)	243 (18.4)	−6.5 (−10.4 to −2.5)	.004
340B status^g^	412 (74.0)	1044 (68.5)	5.5 (1.2 to 9.8)	.02	196 (54.3)	1000 (75.6)	−21.3 (−27.0 to −15.7)	<.001
AMC^h^	88 (15.8)	109 (7.1)	8.7 (5.4 to 11.9)	<.001	25 (6.9)	167 (12.6)	−5.7 (−8.9 to −2.5)	.003
Health system^i^	395 (70.9)	1095 (71.8)	−0.9 (−5.3 to 3.5)	.69	267 (74.0)	940 (71.1)	2.9 (−2.3 to 8.0)	.29

Abbreviations: AMC, Academic Medical Center; HOPD, hospital outpatient department; NA, not applicable; Q, quarter.

^a^ Samples include all facilities where a biologic was administered since quarter of first biosimilar launch in the drug class (Q3 2015 for filgrastim and Q4 2016 for infliximab).

^b^ At least 1 patient received biosimilar administration in practice.

^c^ No patients received biosimilar administration in practice during sample period.

^d^ Comparisons reflect 2-group test of proportions.

^e^ Practice size was determined by the number of physicians billing to a given tax identification number in the 2018 Medicare claims data.

^f^ A practice was deemed multispecialty if less than 80% of the billing physicians to a tax identification number were the same specialty (Table 2).

^g^ 340B status indicates that the hospital associated with the HOPD participated in the 340B Drug Pricing Program as identified through the Health Resources and Services Administration Office of Pharmacy Affairs.

^h^ The hospital associated with the HOPD is an AMC.

^i^ The hospital associated with the HOPD is part of a health system.

### Statistical Analysis

We conducted a number of analyses to examine the extent to which patient, physician, and practice characteristics differed across administrations of biosimilars and originator biologics. Analyses were conducted separately for each drug class.

Mean-comparison *t* tests or tests of proportion were used to compare patient, physician, and practice characteristics across groups. Patient characteristics were compared across patients who ever received a biosimilar vs those who never received a biosimilar. Physician and practice characteristics were compared between physicians and practices that ever administered a biosimilar vs those that did not.

Ordinary least squares multivariable regressions were used to examine the patient, physician, and practice characteristics associated with biosimilar administration. The 3 main regressions included (1) patient and physician characteristics, with an indicator for administration in a hospital outpatient setting; (2) patient, physician, and office characteristics only for administrations within the office setting; and (3) patient, physician, and hospital outpatient characteristics only for administrations within the hospital outpatient setting. All models also controlled for quarter-year and state fixed effects. Patient risk score was excluded from the main regressions, but it was included in sensitivity analyses. In all regressions, robust standard errors were clustered at the patient level.

Sensitivity analyses included comparing patient characteristics in a sample limited to facilities that prescribed at least 1 biosimilar. To examine the sensitivity of results to model specifications, additional analyses included (1) addition of patient risk score covariate; (2) clustering at physician and separately practice level; (3) exclusion of first quarter after initial biosimilar launch; (4) restriction of filgrastim sample to first 9 quarters, the same number as the infliximab sample; (5) restriction to final year or 2 quarters of sample; (6) exclusion of beneficiaries or physicians with low volume; and (7) inclusion only of high volume physicians. The study was conducted using SAS software version 9.4 (SAS Institute Inc) and Stata statistical software version 14.1 (StataCorp). Statistical significance was set at *P* < .05.

## Results

The final filgrastim sample included 25 870 patients (11 857 [45.8%] men; 14 224 [55.0%] aged 65-74 years; 22 617 [87.4%] White individuals) who had 259 178 administrations (79 017 [30.5%] biosimilar administrations) between the quarter 3 2015 launch of filgrastim-sndz and quarter 4 2018. The final infliximab sample included 14 786 patients (4765 [32.2%] men; 8773 [59.3%] aged 65-74 years; 13 467 [91.1%] White) who had 174 973 administrations (9012 [5.2%] biosimilar administrations) between the quarter 4 2016 launch of infliximab-abda and quarter 4 2018 (eFigure 1 and eFigure 2 in the Supplement). Because of missing covariate data, 6725 administrations (2.5%) and 7798 (4.3%) for the filgrastim and infliximab drug classes, respectively, were excluded. Product market share and volume by month appear in eFigure 3 to eFigure 6 in the Supplement. While the market share of filgrastim-sndz has risen to 52% by December 2018, infliximab biosimilars reached 10% of the market by December 2018.

Patients who received at least 1 biosimilar had small differences in demographic and risk characteristics compared with those who had never received a biosimilar, with the exception of age for the filgrastim patients (Table 3). Filgrastim patients who ever received a biosimilar were older than patients who never received a biosimilar (aged ≥75 years: 3950 of 8551 [46.2%] vs 7499 of 17 319 [43.3%]; difference, 2.9 [95% CI, 1.6 to 4.2] percentage points; *P* < .001). The largest differences for both drug classes were among clinical indications. For example, filgrastim patients who had ever received a biosimilar were 3.2 (95% CI, −4.4 to −1.9 percentage points less likely to have neutropenia (percentage points; *P* < .001). Infliximab patients who had ever received a biosimilar were less likely to have Crohn disease (204 of 1514 [13.5%] vs 2228 of 13 272 [16.8%]; difference, −3.3 [95% CI, −5.1 to −1.5] percentage points; *P* = .001) and ulcerative colitis (146 [9.6%] vs 1576 [11.9%]; difference, −2.2 [95% CI, −3.8 to −0.6] percentage points ; *P* = .01) and more likely to have rheumatoid arthritis (1044 [69.0%] vs 8683 [65.4%]; difference, 3.5 [CI, 1.1 to 6.0] percentage points; *P* = .006). Results limiting the sample to patients treated in facilities prescribing at least 1 biosimilar showed similar results (eTable 1 in the Supplement).

**Table 3.** Characteristics of Patients Who Received a Biologic by Receipt of at Least 1 Biosimilar Administration, Filgrastim and Infliximab Drug Classes^a^

Characteristic	Filgrastim sample (Q3 2015-Q4 2018)				Infliximab sample (Q4 2016-Q4 2018)			
	No. (%)		Absolute difference, % (95% CI)^d^	*P* value	No. (%)		Absolute difference, % (95% CI)^d^	*P* value
	≥ 1 (n = 8551)^b^	0 (n = 17 319)^c^			≥1 (n = 1514)^b^	0 (n = 13 272)^c^		
Previous use^e^	957 (11.2)	NA	NA	NA	1088 (71.9)	NA	NA	NA
Age, y								
65-74	4544 (53.1)	9680 (55.9)	−2.8 (−4.0 to −1.5)	<.001	884 (58.4)	7889 (59.4)	−1.1 (−3.7 to 1.6)	.43
≥75	3950 (46.2)	7499 (43.3)	2.9 (1.6 to 4.2)	<.001	590 (39.0)	5044 (38.0)	1.0 (−1.6 to 3.6)	.47
Sex								
Men	3898 (45.6)	7959 (46.0)	−0.4 (−1.7 to 0.9)	.57	479 (31.6)	4286 (32.3)	−0.7 (−3.1 to 1.8)	.61
Women	4653 (54.4)	9360 (54.0)	0.4 (−0.9 to 1.7)	.57	1035 (68.4)	8986 (67.7)	0.7 (−1.8 to 3.1)	.61
Race								
White	7545 (88.2)	15 072 (87.0)	1.2 (0.4 to 2.1)	.006	1392 (91.9)	12 075 (91.0)	1.0 (−0.5 to 2.4)	.21
Black	549 (6.4)	1257 (7.3)	−0.8 (−1.5 to −0.2)	.01	64 (4.2)	676 (5.1)	−0.9 (−1.9 to 0.2)	.14
Other^f^	457 (5.3)	990 (5.7)	−0.4 (−1.0 to 0.2)	.22	58 (3.8)	521 (3.9)	−0.1 (−1.1 to 0.9)	.86
Dual-eligible	712 (8.3)	1733 (10.0)	−1.7 (−2.4 to −0.9)	<.001	77 (5.1)	770 (5.8)	−0.7 (−1.9 to 0.5)	.26
Risk score, mean (SD)^g^	2.54 (1.96)	2.45 (1.91)	−0.09 (−0.14 to −0.03)	.001	1.50 (0.99)	1.46 (0.92)	0.04 (−0.02 to 0.10)	.13
Relevant medical conditions								
Filgrastim-related conditions^h^								
Acute myeloid leukemia	273 (3.2)	457 (2.6)	0.6 (0.1 to 1.0)	.01	NA	NA	NA	NA
Bone marrow								
Harvest	12 (0.1)	11 (0.1)	0.1 (0.0 to 0.2)	.05	NA	NA	NA	NA
Transplantation	247 (2.9)	672 (3.9)	−1.0 (−1.4 to 0.5)	<.001	NA	NA	NA	NA
Neutropenia	3136 (36.7)	6901 (39.8)	−3.2 (−4.4 to −1.9)	<.001	NA	NA	NA	NA
Nonmyeloid malignant neoplasm	6923 (81.0)	13 847 (80.0)	1.0 (0.0 to 2.0)	.06	NA	NA	NA	NA
Infliximab related conditions^i^								
Ankylosing spondylitis	NA	NA	NA	NA	52 (3.4)	577 (4.3)	−0.9 (−1.9 to 0.1)	.10
Crohn disease	NA	NA	NA	NA	204 (13.5)	2228 (16.8)	−3.3 (−5.1 to −1.5)	.001
Plaque psoriasis	NA	NA	NA	NA	111 (7.3)	763 (5.7)	1.6 (0.2 to 3.0)	.01
Arthritis								
Psoriatic	NA	NA	NA	NA	234 (15.5)	1844 (13.9)	1.6 (−0.4 to 3.5)	.10
Rheumatoid	NA	NA	NA	NA	1044 (69.0)	8683 (65.4)	3.5 (1.1 to 6.0)	.006
Ulcerative colitis	NA	NA	NA	NA	146 (9.6)	1576 (11.9)	−2.2 (−3.8 to −0.6)	.01

Abbreviations: NA, not applicable; Q, quarter.

^a^ Samples include all patients who received a biologic since first biosimilar launch in the drug class (Q3 2015 for filgrastim and Q4 2016 for infliximab).

^b^ Patient received at least 1 biosimilar administration during sample period.

^c^ Patient received 0 biosimilar administrations during sample period.

^d^ Comparisons reflect 2-group test of proportions. Mean-comparison (ie, *t*) test used for risk score variables.

^e^ Previous use reflects whether a patient who received at least 1 biosimilar administration also received a nonbiosimilar product in the relevant drug class prior to their first biosimilar administration.

^f^ Other was defined using the Centers for Medicare & Medicaid Services enrollment data as races other than White and Black, which include Asian, Hispanic, North American Native, other, and unknown.

^g^ Risk scores were based on the Department of Health and Human Services Hierarchical Conditions Categories risk adjustment model using claims data from the preceding year.^14^ Sample size for patients with a risk score was smaller because patients had to have 1 year of enrollment prior to first biologic administration (sample size by biosimilar administration for filgrastim was 7564 patients with ≥1 biosimilar administration and 15 448 patients with 0 biosimilar administrations; for infliximab, 1228 patients with ≥1 biosimilar administration and 10 694 patients with 0 biosimilar administrations).

^h^ Conditions are not mutually exclusive. Filgrastim-related conditions are chemotherapy treatment with acute myeloid leukemia; bone marrow harvest; bone marrow transplant following chemotherapy in patients with nonmyeloid malignant neoplasms; congenital, cyclic, and idiopathic neutropenia; and chemotherapy treatment with nonmyeloid malignant neoplasm.

^i^ Conditions are not mutually exclusive.

Of the 14 971 physicians who administered filgrastim, 5713 (38.2%) administered at least 1 biosimilar, whereas only 1106 (10.7%) of the 10 318 physicians in the infliximab sample administered a biosimilar (Table 1). Physicians who ever administered a biosimilar administered them frequently: among these physicians, a mean (SD) of 76.6% (31.6%) and 49.4% (34.4%) of filgrastim and infliximab administrations, respectively, were biosimilars. Additionally, these physicians were more likely to administer a high volume of biologic medications relative to physicians who never administered a biosimilar (filgrastim, 1395 [24.5%] vs 1726 [18.6%]; difference, 5.8 [95% CI, 4.5 to 7.1] percentage points; *P* < .001; infliximab, 352 [31.8%] vs 1616 [17.5%]; difference, 14.3 [95% CI, 11.9 to 16.8] percentage points; *P* < .001). Physician years in practice and sex were similar by biosimilar administration; however, physicians who administered infliximab biosimilars were much more likely to practice in a hospital-owned practice (453 [41.0%] vs 2679 [29.1%]; difference, 11.9 [95% CI, 8.8 to 14.9] percentage points; *P* < .001). Physician specialty was also different by biosimilar uptake.

More than half of all administrations for both drug classes took place in an office setting, 137 380 of 259 178 (53.0%) and 110 586 of 174 974 (63.2%) for the filgrastim and infliximab drug classes, respectively. If a practice or hospital outpatient department ever had a biosimilar administration, a large portion of its administrations were biosimilars (mean [SD] biosimilar administration rate for filgrastim, practices: 50.9% [32.2%]; hospital outpatient departments, 59.3% [31.5%]; infliximab, practices: 33.2% [30.6%]; hospital outpatient departments, 37.4% [29.0%]) (Table 2). Compared with practices and hospital outpatient departments that never administered a biosimilar, those that did were less likely to be small (practices with ≤5 physicians: 366 of 636 [57.5%] vs 150 of 327 [46.0%]; difference, −11.5 [95% CI, −18.2 to −4.9] percentage points, *P* = .001; hospitals with ≤50 beds: 425 of 1525 [27.9%] vs 76 of 558 [13.6%]; difference, −14.2 [−17.9 to −10.6] percentage points; *P* < .001). Additional differences between hospital outpatient settings that ever or never adopted a biosimilar were inconsistent across drug classes. Hospital outpatient departments that administered filgrastim biosimilars were more likely to be not-for-profit, have 340B status, or be an academic medical center than those that did not (not-for-profit: 461 [82.8%] vs 1041 [68.3%]; difference, 14.5 [95% CI, 10.6 to 18.4] percentage points; *P* < .001; 340B status: 412 [74.0%] vs 1044 [68.5%]; difference, 5.5 [95% CI, 1.2 to 9.8] percentage points; *P* = .02; academic medical center: 88 [15.8%] vs 109 [7.1%]; difference, 8.7 [95% CI, 5.4 to 11.9]; *P* < .001), but these characteristics were less likely for hospital outpatient departments that administered infliximab biosimilars (for-profit: 60 [16.6%] vs 89 [6.7%]; difference, 9.9 [95% CI, −5.8 to 14.0] percentage points; *P* < .001; 340B status: 196 [54.3%] vs 1000 [75.6%]; difference, −21.3 [95% CI, −27.0 to −15.7] percentage points; *P* < .001; academic medical center: 25 [6.9%] vs 167 [12.6%]; difference, −5.7 [95% CI, −8.9 to −2.5]; *P* = .003).

**Figure 1.**
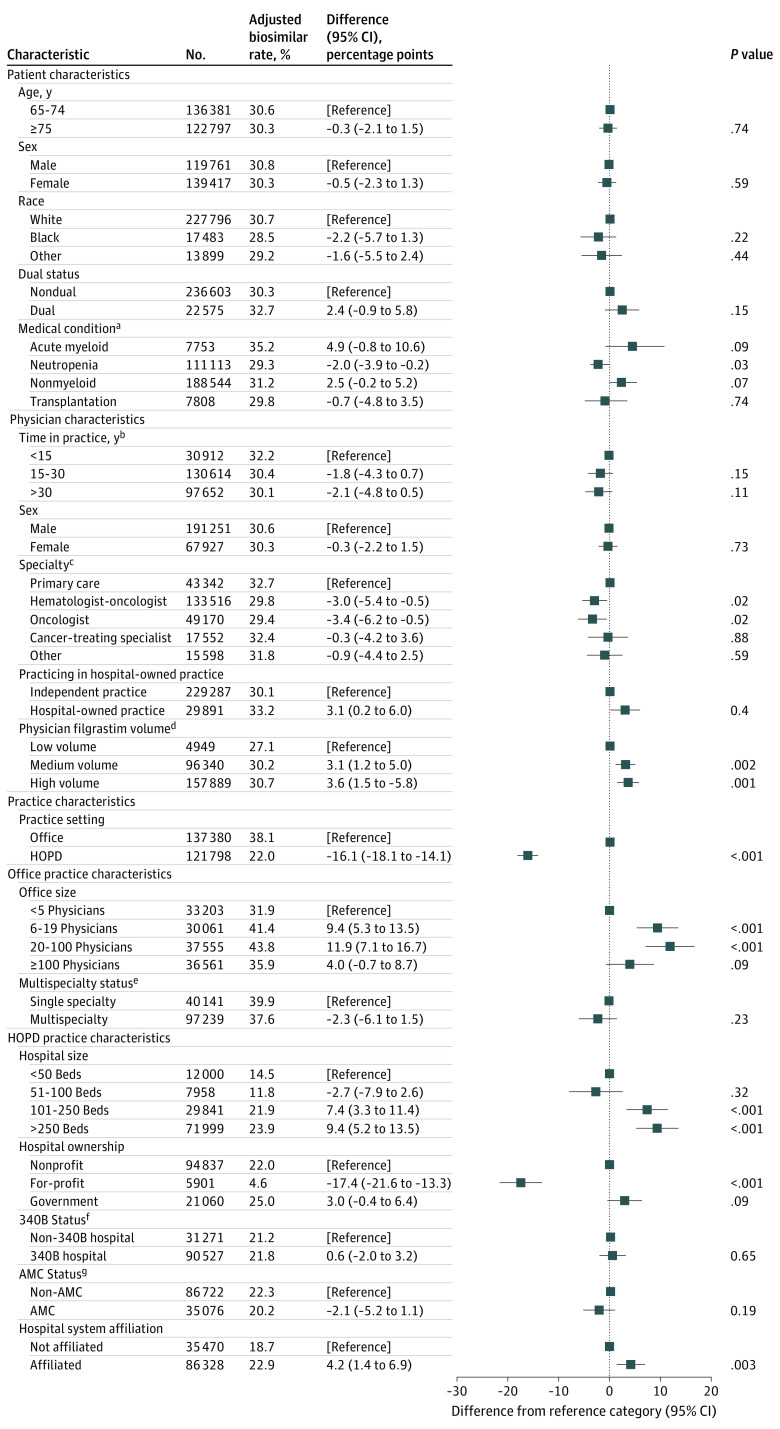
Association Between Filgrastim Biosimilar Administration and Patient, Physician, and Practice Characteristics Results were obtained from ordinary least-squares regressions of indicator of biosimilar administration on listed covariates. Patient, physician, and practice setting results are from regression with patient and physician covariates and practice setting indicator. Office practice results are from regression with office covariates and physician and practice covariates and includes only administrations in an office practice. Hospital outpatient department (HOPD) results are from regression with HOPD covariates and physician and practice covariates and includes only administrations in an HOPD. All models also included year-quarter and state fixed effects and clustered robust standard errors at the patient level. AMC indicates academic medical center. ^a^Reference categories are patients without a condition. Filgrastim-related conditions are not mutually exclusive and include chemotherapy treatment with acute myeloid leukemia; bone marrow transplant following chemotherapy in patients with nonmyeloid malignant neoplasms; congenital, cyclic, and idiopathic neutropenia; and chemotherapy treatment with nonmyeloid malignant neoplasm. Bone marrow harvest indication excluded due to small sample size. ^b^Years in practice indicates years since completion of medical school. ^c^Other cancer-related specialties include gastroenterology, gynecology, proctology, urology, nephrology, endocrinology, and hematology. ^d^Low, medium, and high volume defined as 1 or fewer, more than 1 to 5, and more than 5 average monthly filgrastim administrations. ^e^A practice was deemed multispecialty if less than 80% of the billing physicians to a tax identification number were the same specialty. Specialty designations appear in Table 2. ^f^340B status indicates that the hospital associated with the HOPD participated in the 340B Drug Pricing Program as identified through the Health Resources and Services Administration Office of Pharmacy Affairs. ^g^The hospital associated with the HOPD is an AMC.

In adjusted filgrastim analyses (Figure 1), neutropenia was the only patient characteristic associated with whether an administration was a biosimilar (adjusted difference, −2.0 [95% CI, −3.9 to −0.2] percentage points; *P* = .03). Physician characteristics, including specialty and biologic drug volume, were associated with biosimilar administrations. Relative to primary care physicians, filgrastim administered by hematologist-oncologists or oncologists were less likely to be a biosimilar (hematologist-oncologist: difference, −3.0 [95% CI, −5.4 to −0.5] percentage points; *P* = .02; oncologist: −3.4 [95% CI, −6.2 to −0.5] percentage points; *P* = .02). High-volume physicians were 3.6 (95% CI, 1.5 to 5.7) percentage points more likely to administer a filgrastim biosimilar relative to low-volume physicians (*P* = .001). Administrations in the hospital outpatient setting were 16.1 (95% CI, 14.1 to 18.1) percentage points less likely to be a biosimilar (*P* < .001). Within an office or hospital outpatient setting, biosimilars were more likely to be administered in larger offices or hospital outpatient departments and were 17.4 (95% CI, 13.3 to 21.6) percentage points less likely to be administered in a for-profit setting than a not-for-profit setting (*P* < .001).

**Figure 2.**
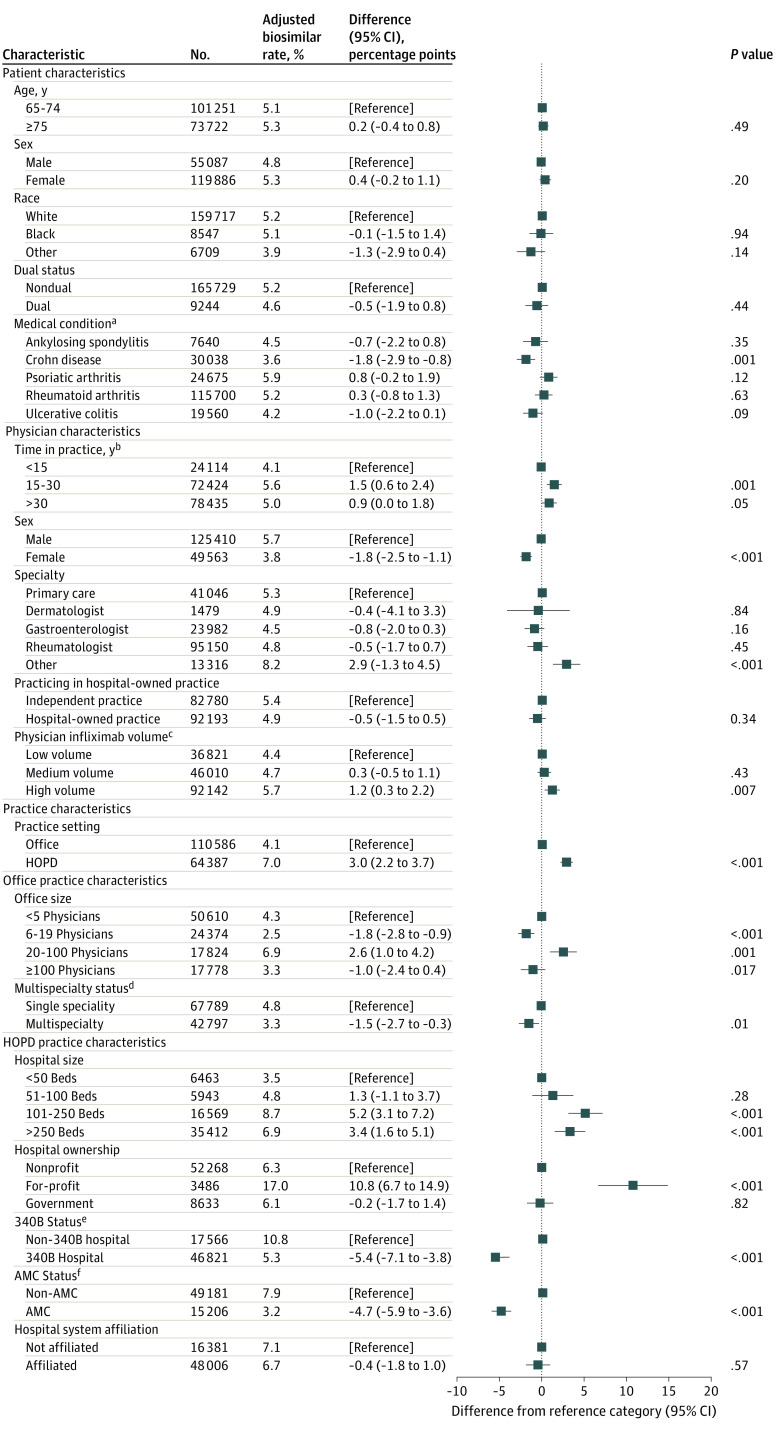
Association Between Infliximab Biosimilar Administration and Patient, Physician and Practice Characteristics Results were obtained from ordinary least-squares regressions of indicator of biosimilar administration on listed covariates. Patient, physician, and practice setting results are from regression with patient and physician covariates and practice setting indicator. Office practice results are from regression with office covariates and physician and practice covariates and includes only administrations in an office practice. Hospital outpatient department (HOPD) results are from regression with HOPD covariates and physician and practice covariates and includes only administrations in an HOPD. All models also included year-quarter and state fixed effects and clustered robust standard errors at the patient level. AMC indicates academic medical center. ^a^Reference categories are patients without a condition. Infliximab-related conditions are not mutually exclusive. ^b^Time in practice indicates years since completion of medical school. ^c^Low, medium, and high volume defined as 1 or fewer, more than 1 to 2, and more than 2 average monthly infliximab administrations. ^d^A practice was deemed multispecialty if less than 80% of the billing physicians to a tax identification number were the same specialty. Specialty designations appear in Table 2. ^e^340B status indicates that the hospital associated with the HOPD participated in the 340B Drug Pricing Program as identified through the Health Resources and Services Administration Office of Pharmacy Affairs. ^f^The hospital associated with the HOPD is an AMC.

In adjusted infliximab analyses (Figure 2), the only patient characteristic associated with a biosimilar administration was presence of Crohn disease; these patients were 1.8 (95% CI, 0.8 to 2.9) percentage points less likely to receive a biosimilar (*P* = .001). Numerous physician characteristics were associated with biosimilar administrations, including being male (male vs female physician: difference, 1.8 [95% CI, 1.1 to 2.5] percentage points; *P* < .001), more years of practice (15-30 years vs ≤15 years: difference, 1.5 [95% CI, 0.6 to 2.4] percentage points; *P* = .001), and higher prescribing volume (high vs low volume: difference, 1.2 [95% CI, 0.3 to 2.2] percentage points; *P* = .007). The factor with the largest association with biosimilar administrations was the setting. Compared with the office setting, administrations in the hospital outpatient setting were 3.0 (95% CI, 2.2 to 3.7) percentage points more likely to be a biosimilar (*P* < .001). Infliximab biosimilar administrations were more likely to take place in larger offices or hospital outpatient departments. Numerous other hospital outpatient setting characteristics were associated with biosimilar administrations. Sensitivity analyses, presented in eTable 2 to eTable 9 in the Supplement, yielded similar results.

## Discussion

The decision to administer a biosimilar rather than an originator biologic may be influenced by characteristics of patients, physicians, or the setting of administration. In this study of factors associated with biosimilar uptake in the Medicare fee-for-service population, patient characteristics were weakly associated with biosimilar uptake, while few physician characteristics, including higher product volume and physician specialty, were associated with biosimilar use. The practice setting of an administration had the strongest association with whether a patient received a biosimilar; however, the direction of the association differed by drug class. In adjusted analyses, a patient in the hospital outpatient setting was 16 percentage points or 42% less likely to receive a filgrastim biosimilar than a patient in an office setting but 3 percentage points or 73% more likely to receive an infliximab biosimilar. For administrations in a hospital outpatient setting, ownership status was also associated with biosimilar utilization but again in opposite directions by drug class. Patients were 17 percentage points less likely and 11 percentage points more likely to receive filgrastim and infliximab biosimilars, respectively, in for-profit vs not-for-profit hospital outpatient departments.

This work supports previous evidence from studies of filgrastim, which suggest that patient characteristics are not strongly associated with biosimilar use.^15^ Adjusted analyses controlling for patient and practice characteristics found that both physician specialty^15,16^ and physician prescribing volume^15^ of a given drug class remained strongly associated with biosimilar use. Although results were not consistent in the filgrastim drug class, physicians with a higher volume of prescriptions were generally more likely to prescribe biosimilar products. This could be because of limited awareness or comfort with biosimilars among physicians with lower rates of prescribing.^19^ How to encourage use of biosimilars among this latter group of physicians is an open question.

To our knowledge, this is the first study to document that practice setting is associated with infliximab biosimilar administrations, with uptake significantly higher in the hospital outpatient setting than the physician office setting. The association in practice setting uptake was inverted for filgrastim; there was a higher rate of biosimilar uptake in physician practices than in hospital outpatient departments, a result that was consistent with previous work. Importantly, this study demonstrates that factors associated with biosimilar use may differ across drug classes, and therefore, each drug class may require different interventions to promote use.

The fact that practice setting was consistently associated with prescribing when controlling for patient and physician characteristics suggests that physician practices or hospital outpatient departments may play an important role in steering prescribers toward certain medications. This could occur either explicitly or implicitly, for instance by limiting products on a formulary or purchasing schedule. Practices and hospital outpatient departments may want to steer physicians toward certain products when they can earn higher profits on those products. For the average practice and hospital outpatient department, the profit from administering a biologic and biosimilar to a Medicare beneficiary is the same, given that Medicare reimburses the medication at their respective average sales price plus 6% of the reference biologic’s average sales price. As originator biologics are generally more expensive than biosimilars, it is possible that practices that receive the largest discounts may find originator biologics more profitable if they are able to get a lower net price for these products. Traditionally, hospital outpatient departments are able to receive larger discounts relative to smaller office practices due to their high-volume purchasing, which could be an explanation for slower filgrastim-sndz uptake in the hospital outpatient setting. However, this fact may not hold for infliximab: the manufacturer of the originator (Johnson and Johnson) has pursued unique contracting mechanisms to discourage biosimilar usage, which could have a large financial impact on both small and large organizations.

Other proposed explanations for differential uptake between practice settings have included patients with more severe illness selecting 1 setting over another and more complicated purchasing processes in the hospital outpatient setting, leading to slower adoption. For the former, we found that practice setting remained associated with biosimilar use even when controlling for patient and physician characteristics, suggesting this is not a complete explanation. For the latter, the fact that infliximab uptake was actually higher in the hospital outpatient setting suggests that while complex purchasing agreements may play a role, they are also not the complete story.

As this work demonstrates, adoption of biosimilars in Medicare has been uneven across different products. The uptake of filgrastim-sndz has risen consistently over time, reaching 52% of the market by December 2018. However, the uptake of infliximab biosimilars has been significantly slower, reaching 10% of the market by December 2018. This pattern is consistent with the European experience, where infliximab uptake was significantly slower than filgrastim,^6^ and represents a lost opportunity for cost savings. There are a number of potential explanations for the slower uptake of infliximab biosimilars. Given that infliximab is used for chronic conditions, physicians may be less willing to switch patients who are doing well on the originator brand to a biosimilar.^10^ Furthermore, financial incentives for infliximab and its biosimilars are likely to differ from filgrastim. The unique contracting mechanisms used by the manufacturer of originator infliximab (ie, Johnson and Johnson) have included exclusionary contracts and purchasing bundles with health care professionals, health care systems, and insurers to allegedly block biosimilar competition. These contracting mechanisms include so-called rebate traps, which withdraw the rebates payers and clinicians get for prescribing biologics if patients switch to a biosimilar.^10^ Future work should investigate how contracting mechanisms might encourage, rather than discourage, the use of more efficient alternatives, such as biosimilars.

### Limitations

This study has several limitations. First, it focuses on the Medicare population and thus misses individuals who are privately insured, for whom uptake patterns could be different. Second, this study cannot determine whether there is a causal link between patient, physician, and practice characteristics and biosimilar administration, although the analysis controls for many observable confounders. Third, this study focuses on the short-term uptake of 3 biosimilars in 2 drug classes. It is possible that long-term trends as well as trends for biosimilars in other drug classes will differ.

## Conclusions

In this cross-sectional study of factors associated with the uptake of the first 3 biosimilars launched in Medicare, the practice setting (ie, office vs hospital outpatient department) and hospital outpatient ownership status had the strongest association with biosimilar use. Surprisingly, the direction of the associations between practice setting and uptake was opposite for the 2 drug classes. Further research is needed to understand the reasons underlying differences in biosimilar uptake across practice settings for the filgrastim and infliximab drug classes.

## Notes

Supplement. **eFigure 1.** Generation of the Analytic Sample, Filgrastim **eFigure 2.** Generation of the Analytic Sample, Infliximab **eFigure 3.** Percentage of Filgrastim Product Volume and Total Filgrastim Volume, by Month **eFigure 4.** Percentage of Infliximab Product Volume and Total Infliximab Volume, by Month **eFigure 5.** Percentage of Filgrastim Product Volume and Total Filgrastim Volume in the Hospital and Office Settings, by Month **eFigure 6.** Percentage of Infliximab Product Volume and Total Infliximab Volume in the Hospital and Office Settings, by Month **eAppendix.** Physician Attribution **eTable 1.** Characteristics of Patients Who Received a Biologic by Receipt of at Least 1 Biosimilar Administration in Facilities With at Least 1 Biosimilar Administration, Filgrastim and Infliximab Drug Classes **eTable 2.** Association Between Filgrastim Biosimilar Administration and Patient, Physician and Practice Characteristics With Patient Risk Score **eTable 3.** Association Between Filgrastim Biosimilar Administration and Patient, Physician and Practice Characteristics, Model Specification Sensitivities **eTable 4.** Association Between Filgrastim Biosimilar Administration and Patient, Physician and Practice Characteristics, Modifying Time Periods **eTable 5.** Association Between Filgrastim Biosimilar Administration and Patient, Physician and Practice Characteristics, Inclusion/Exclusion Based on Volume **eTable 6.** Association Between Infliximab Biosimilar Administration and Patient, Physician and Practice Characteristics With Patient Risk Score **eTable 7.** Association Between Infliximab Biosimilar Administration and Patient, Physician and Practice Characteristics, Model Specification Sensitivities **eTable 8.** Association Between Infliximab Biosimilar Administration and Patient, Physician and Practice Characteristics, Modifying Time Periods **eTable 9.** Association Between Infliximab Biosimilar Administration and Patient, Physician and Practice Characteristics, Inclusion/Exclusion Based on Volume
