# Supplementary material for: Physician, Practice, and Patient Characteristics Associated With Biosimilar Use in Medicare Recipients
Source: JAMA Netw Open. 2021 Jan 27;4(1):e2034776. doi: 10.1001/jamanetworkopen.2020.34776 (PMC7841457; doi:10.1001/jamanetworkopen.2020.34776)
Supplement: Supplement. — eFigure 1. Generation of the Analytic Sample, Filgrastim eFigure 2. Generation of the Analytic Sample, Infliximab eFigure 3. Percentage of Filgrastim Product Volume and Total Filgrastim Volume, by Month eFigure 4. Percentage of Infliximab Product Volume and Total Infliximab Volume, by Month eFigure 5. Percentage of Filgrastim Product Volume and Total Filgrastim Volume in the Hospital and Office Settings, by Month eFigure 6. Percentage of Infliximab Product Volume and Total Infliximab Volume in the Hospital and Office Settings, by Month eAppendix. Physician Attribution eTable 1. Characteristics of Patients Who Received a Biologic by Receipt of at Least 1 Biosimilar Administration in Facilities With at Least 1 Biosimilar Administration, Filgrastim and Infliximab Drug Classes eTable 2. Association Between Filgrastim Biosimilar Administration and Patient, Physician and Practice Characteristics With Patient Risk Score eTable 3. Association Between Filgrastim Biosimilar Administration and Patient, Physician and Practice Characteristics, Model Specification Sensitivities eTable 4. Association Between Filgrastim Biosimilar Administration and Patient, Physician and Practice Characteristics, Modifying Time Periods eTable 5. Association Between Filgrastim Biosimilar Administration and Patient, Physician and Practice Characteristics, Inclusion/Exclusion Based on Volume eTable 6. Association Between Infliximab Biosimilar Administration and Patient, Physician and Practice Characteristics With Patient Risk Score eTable 7. Association Between Infliximab Biosimilar Administration and Patient, Physician and Practice Characteristics, Model Specification Sensitivities eTable 8. Association Between Infliximab Biosimilar Administration and Patient, Physician and Practice Characteristics, Modifying Time Periods eTable 9. Association Between Infliximab Biosimilar Administration and Patient, Physician and Practice Characteristics, Inclusion/Exclusion Based on Volume [file jamanetwopen-e2034776-s001.pdf]

Supplemental Online Content

Dean EB, Johnson P, Bond AM. Physician, practice, and patient characteristics associated with biosimilar use in Medicare recipients. *JAMA Network Open*. 2021;4(1):e2034776. doi:10.1001/jamanetworkopen.2020.34776

eFigure 1. Generation of the Analytic Sample, Filgrastim
eFigure 2. Generation of the Analytic Sample, Infliximab
eFigure 3. Percentage of Filgrastim Product Volume and Total Filgrastim Volume, by Month
eFigure 4. Percentage of Infliximab Product Volume and Total Infliximab Volume, by Month
eFigure 5. Percentage of Filgrastim Product Volume and Total Filgrastim Volume in the Hospital and Office Settings, by Month
eFigure 6. Percentage of Infliximab Product Volume and Total Infliximab Volume in the Hospital and Office Settings, by Month
eAppendix. Physician Attribution
eTable 1. Characteristics of Patients Who Received a Biologic by Receipt of at Least 1 Biosimilar Administration in Facilities With at Least 1 Biosimilar Administration, Filgrastim and Infliximab Drug Classes
eTable 2. Association Between Filgrastim Biosimilar Administration and Patient, Physician and Practice Characteristics With Patient Risk Score
eTable 3. Association Between Filgrastim Biosimilar Administration and Patient, Physician and Practice Characteristics, Model Specification Sensitivities
eTable 4. Association Between Filgrastim Biosimilar Administration and Patient, Physician and Practice Characteristics, Modifying Time Periods
eTable 5. Association Between Filgrastim Biosimilar Administration and Patient, Physician and Practice Characteristics, Inclusion/Exclusion Based on Volume
eTable 6. Association Between Infliximab Biosimilar Administration and Patient, Physician and Practice Characteristics With Patient Risk Score
eTable 7. Association Between Infliximab Biosimilar Administration and Patient, Physician and Practice Characteristics, Model Specification Sensitivities
eTable 8. Association Between Infliximab Biosimilar Administration and Patient, Physician and Practice Characteristics, Modifying Time Periods
eTable 9. Association Between Infliximab Biosimilar Administration and Patient, Physician and Practice Characteristics, Inclusion/Exclusion Based on Volume

This supplemental material has been provided by the authors to give readers additional information about their work.

© 2021 Dean EB et al. *JAMA Network Open.*

eFigure 1. Generation of the Analytic Sample, Filgrastim

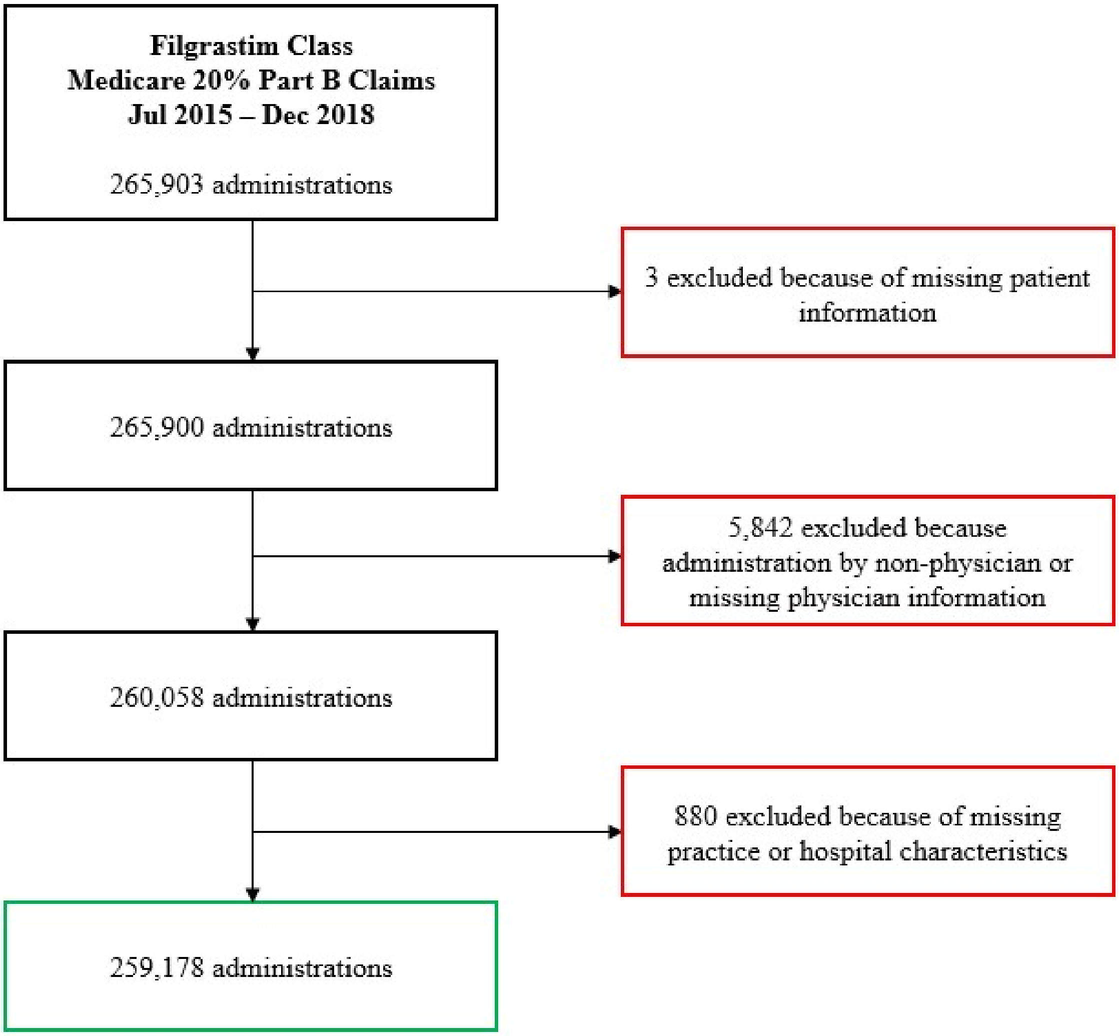

© 2021 Dean EB et al. *JAMA Network Open.*

eFigure 2. Generation of the Analytic Sample, Infliximab

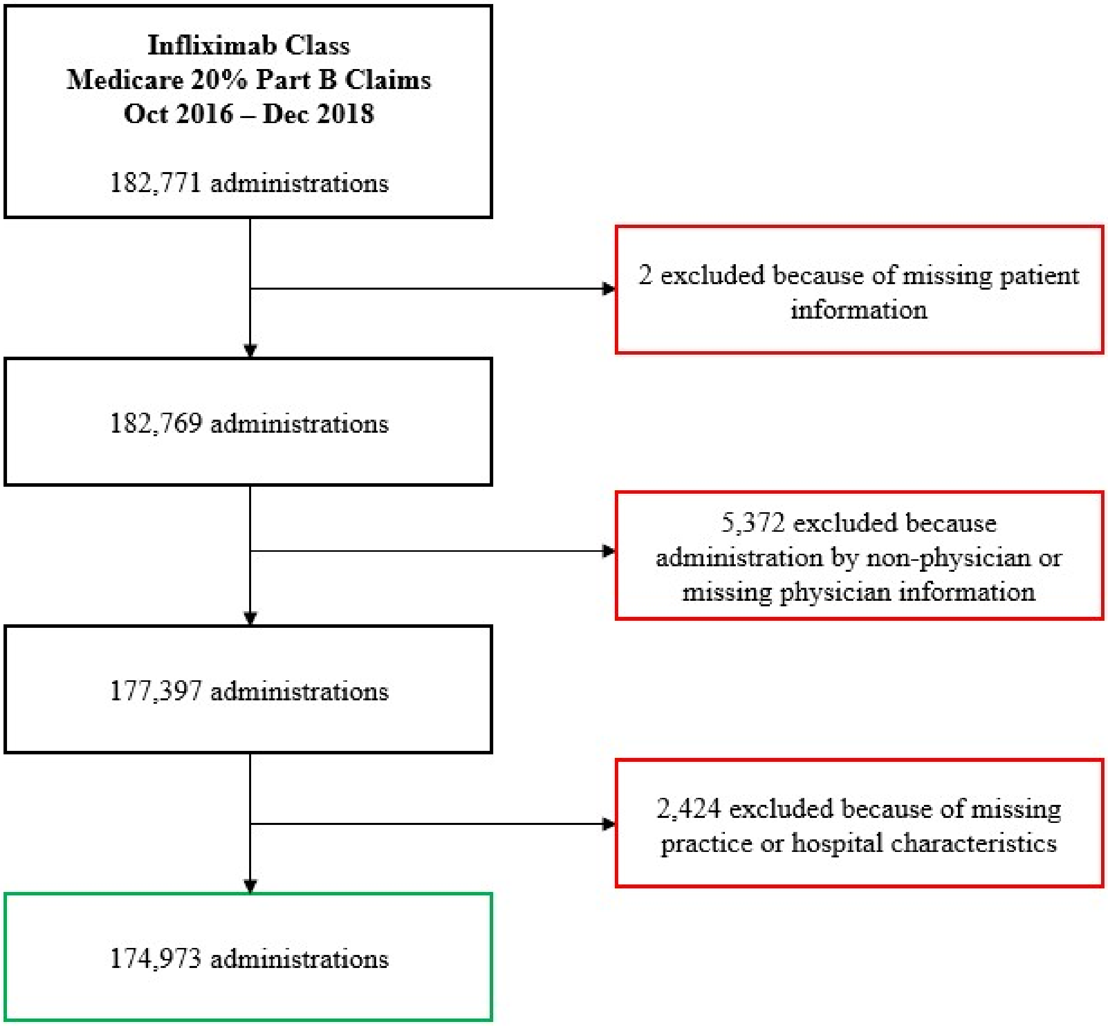

© 2021 Dean EB et al. *JAMA Network Open.*

eFigure 3. Percentage of Filgrastim Product Volume and Total Filgrastim Volume, by Month

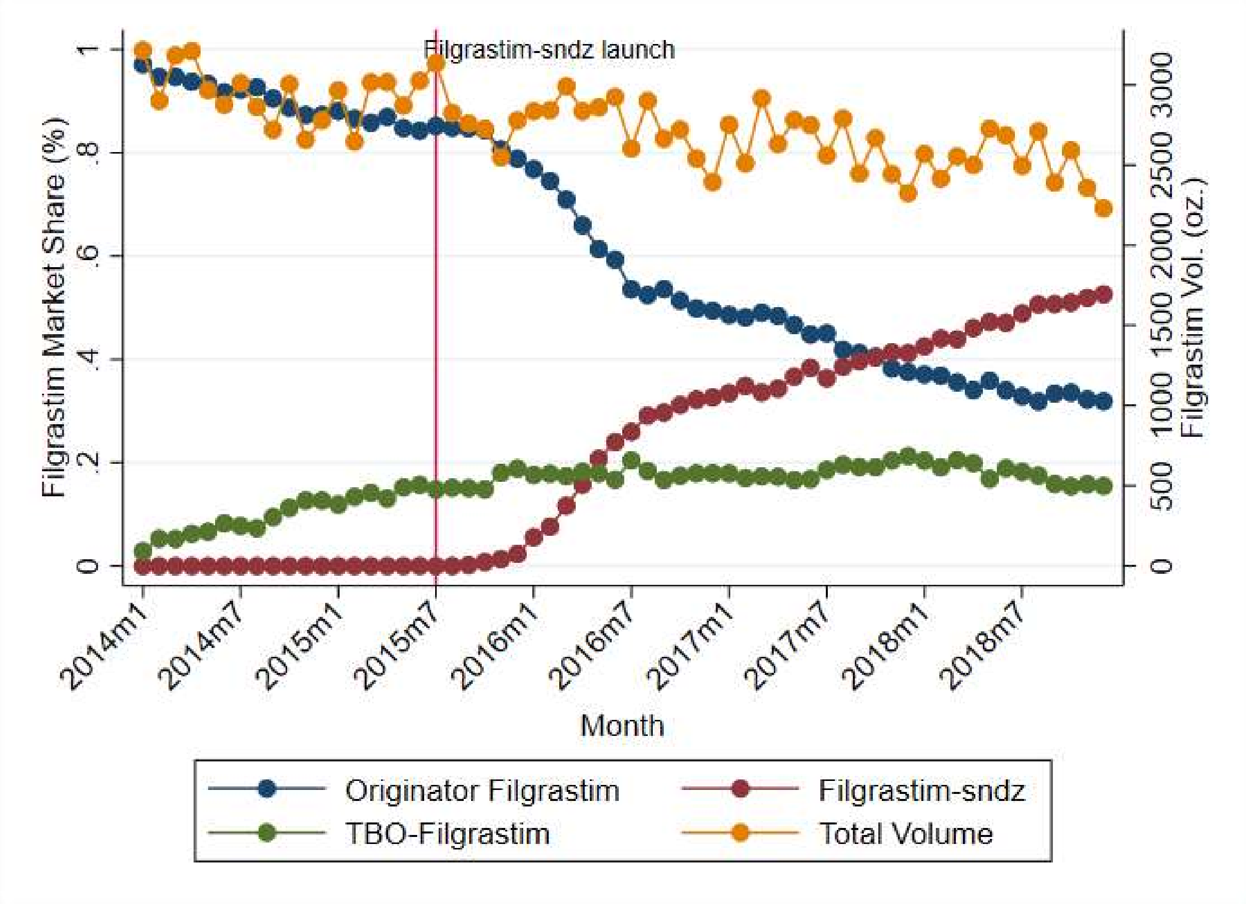

Red line denotes the quarter of the filgrastim-sndz launch in Medicare.

© 2021 Dean EB et al. *JAMA Network Open.*

eFigure 4. Percentage of Infliximab Product Volume and Total Infliximab Volume, by Month

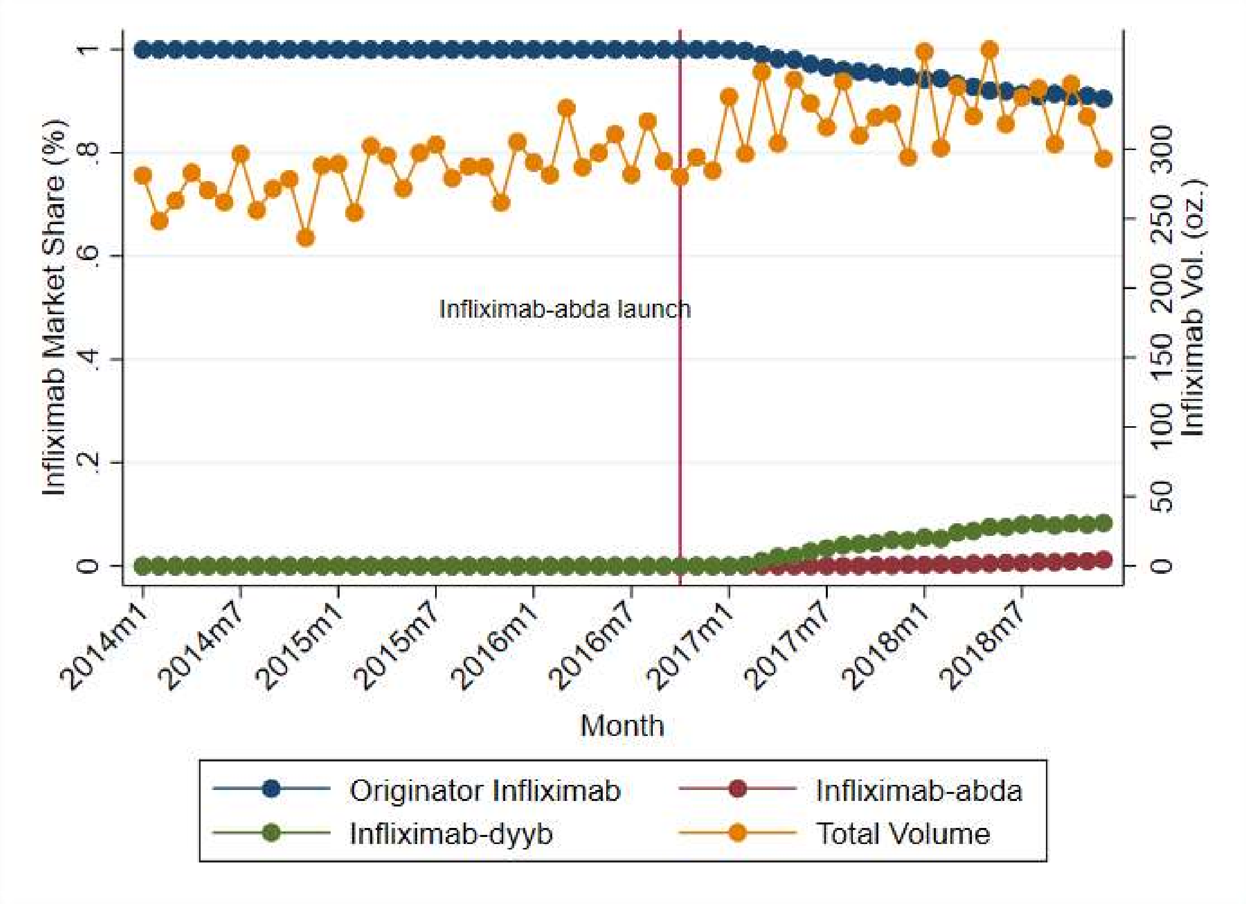

Red line denotes the quarter of the infliximab-abda launch in Medicare.

© 2021 Dean EB et al. *JAMA Network Open.*

eFigure 5. Percentage of Filgrastim Product Volume and Total Filgrastim Volume in the Hospital and Office Settings, by Month

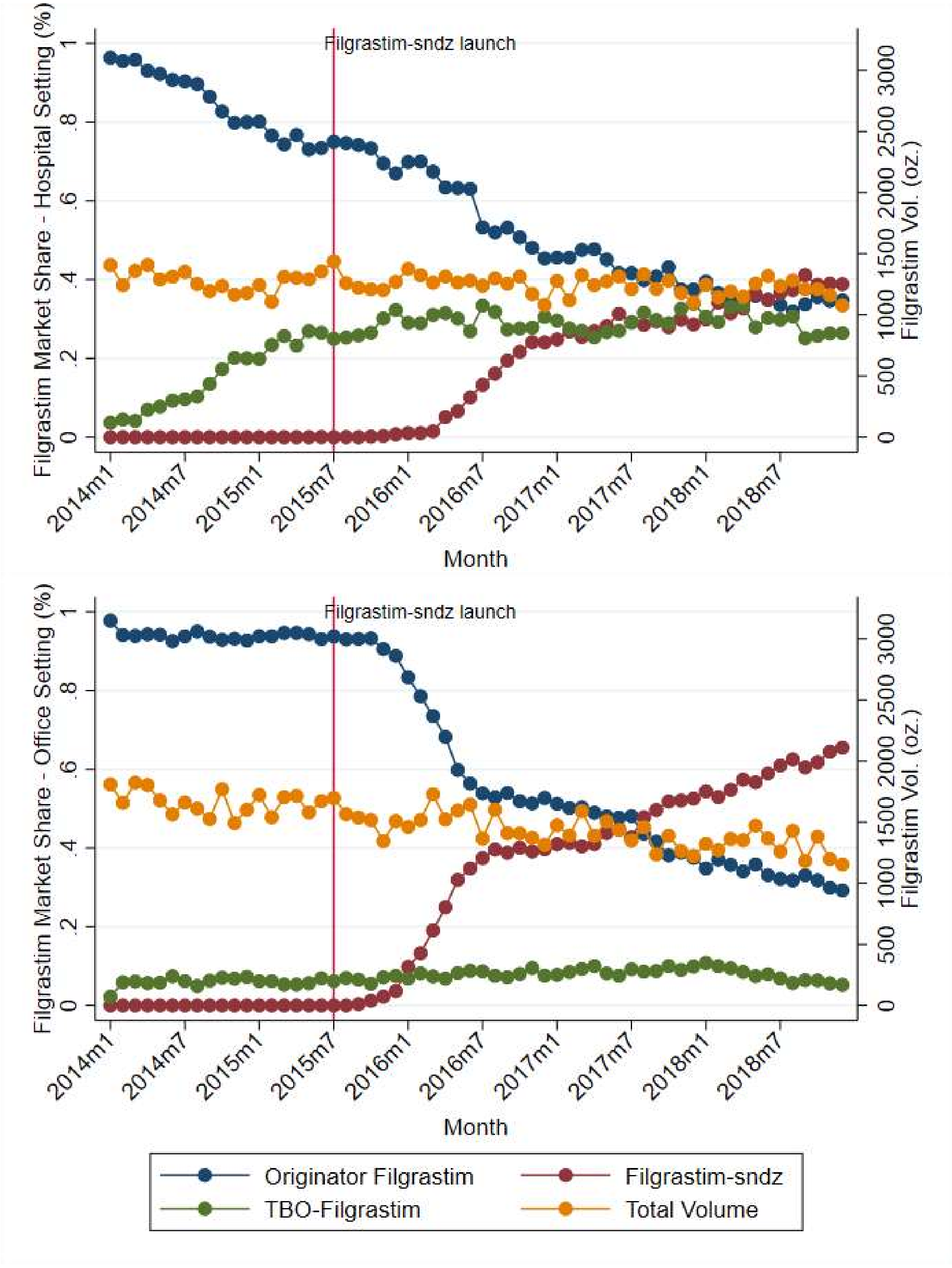

© 2021 Dean EB et al. *JAMA Network Open.*

eFigure 6. Percentage of Infliximab Product Volume and Total Infliximab Volume in the Hospital and Office Settings, by Month

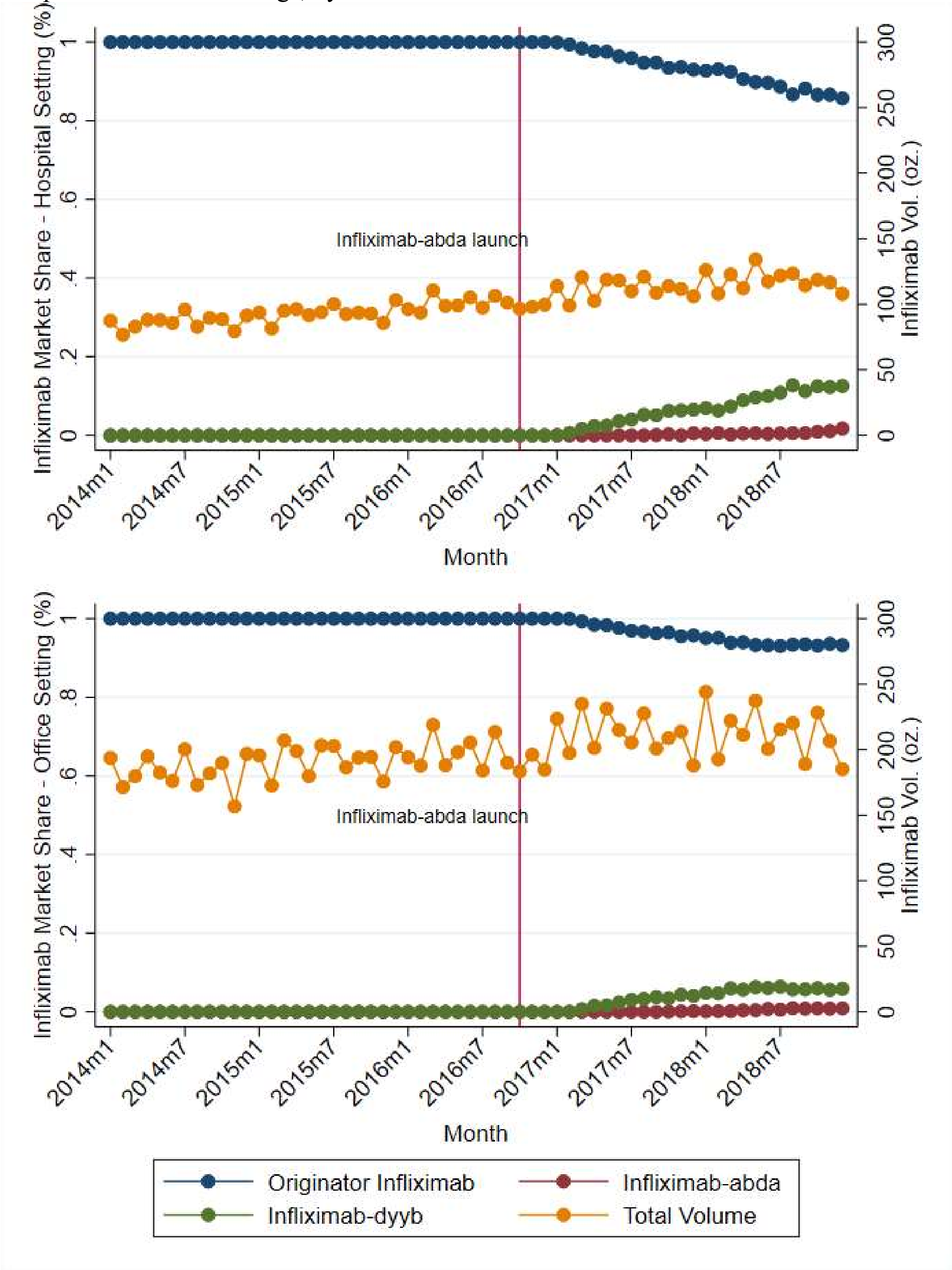

eAppendix. Physician Attribution

Physicians were attributed to patients in a four step process:

1. The referring physician NPI associated with a relevant biologic HCPCS code was selected from the carrier base claim file (physician office setting). The prescribing NPI associated with a relevant biologic HCPCS code was selected from the Outpatient Encounter file (HOPD setting).
2. If a patient had a missing NPI for one biologic administration, but had a referring/billing NPI on a previous claim, the prior NPI was assigned to the administration with a missing NPI.
3. Physician specialty was obtained from the 2018 Physician Compare database, or from 2017-2014 Physician Compare databases when a relevant NPI was not available in the 2018 Physician Compare database. Relevant specialties likely to prescribe our biologic products of interest were flagged. For the filgrastim drug class, these specialties were: hematologist-oncologist, oncologist, cancer-treating specialties (gastroenterology, gynecology, proctology, urology, nephrology, endocrinology, and hematology), and primary care. For the infliximab drug class, these specialties were rheumatology, gastroenterology, dermatology, and primary care.
4. When a biologic administration was attributed to an NPI associated with a nurse or non-relevant specialty and a previous administration for the same product was attributed to an NPI with relevant specialty, the administrations assigned to a nurse or non-relevant specialty were reassigned to the most recent previous NPI with a relevant specialty. 8.3% of administrations were reassigned for filgrastim and 7.0% for infliximab.

© 2021 Dean EB et al. *JAMA Network Open.*

eTable 1. Characteristics of Patients Who Received a Biologic by Receipt of at Least 1 Biosimilar Administration in Facilities With at Least 1 Biosimilar Administration, Filgrastim and Infliximab Drug Classes[a]

	**Filgrastim sample (Q3 2015 – Q4 2018)**			**Infliximab sample (Q4 2016 – Q4 2018)**		
	**At least one[a]**	**None[b]**	**Difference in Means ((95% CI) [P-value])[c]**	**At least one[a]**	**None[b]**	**Difference in Means ((95% CI) [P-value])[c]**
**N[d]**	8,253	6,308		1,415	2,412	
**Previous Use (%)[e]**	812 (9.8)	-		993 (70.2)	-	
**Age (%)**						
65-74	4,377 (53.0)	3,531 (56.0)	-2.9 (-4.6 to -1.3) [P<0.001]	828 (58.5)	1,427 (59.2)	-0.6 (-3.9 to 2.6) [P=0.695]
75+	3,821 (46.3)	2,726 (43.2)	3.1 (1.5 to 4.7) [P<0.001]	549 (38.8)	930 (38.6)	0.2 (-3.0 to 3.4) [P=0.882]
**Sex (%)**						
Male	3,756 (45.5)	2,955 (46.8)	-1.3 (-3.0 to 0.3) [P=0.109]	444 (31.4)	765 (31.7)	-0.3 (-3.4 to 2.7) [P=0.828]
Female	4,497 (54.5)	3,353 (53.2)	1.3 (-0.3 to 3.0) [P=0.109]	971 (68.6)	1,647 (68.3)	0.3 (-2.7 to 3.4) [P=0.828]
**Race (%)**						
White	7,269 (88.1)	5,490 (87.0)	1.0 (0.0 to 2.1) [P=0.058]	1,300 (91.9)	2,187 (90.7)	1.2 (-0.6 to 3.0) [P=0.207]
Black	542 (6.6)	465 (7.4)	-0.8 (-1.6 to 0.0) [P=0.058]	62 (4.4)	111 (4.6)	-0.2 (-1.6 to 1.1) [P=0.751]
Other	442 (5.4)	353 (5.6)	-0.2 (-1.0 to 0.5) [P=0.527]	53 (3.7)	114 (4.7)	-1.0 (-2.3 to 0.3) [P=0.152]
**Dual-Eligible (%)**	686 (8.3)	563 (8.9)	-0.6 (-1.5 to -0.3) [P=0.191]	74 (5.2)	128 (5.3)	-0.1 (-1.5 to 1.4) [P=0.918]
**Risk score (Mean (SD))[f]**	2.5	2.6	-0.1 (-0.2 to -0.1) [P<0.001]	1.5	1.4	0.1 (-0.0 to 0.1) [P=0.121]
**Relevant medical conditions**						
Filgrastim related conditions[g]						
Acute myeloid leukemia	6,731 (81.6)	5,121 (81.2)	0.4 (-0.9 to 1.7) [P=0.564]			
Bone marrow harvest	260 (3.2)	189 (3.0)	0.2 (-0.4 to 0.7) [P=0.594]			
Bone marrow transplant	224 (2.7)	254 (4.0)	-1.3 (-1.9 to -0.7) [P<0.001]			
Neutropenia	9 (0.1)	8 (0.1)	-0.0 (-0.1 to 0.1) [P=0.756]			
Nonmyeloid malignancy	2,988 (36.2)	2,395 (38.0)	-1.8 (-3.3 to -0.2) [P=0.029]			
Infliximab related conditions[h]						
Ankylosing Spondylitis				187 (13.2)	356 (14.8)	-1.5 (-3.8 to 0.7) [P=0.186]
Crohn's Disease				141 (10.0)	240 (10.0)	0.0 (-2.0 to 2.0) [P=0.989]
Plaque Psoriasis				977 (69.0)	1,688 (70.0)	-0.9 (-4.0 to 2.1) [P=0.543]

© 2021 Dean EB et al. *JAMA Network Open.*

Psoriatic Arthritis				50 (3.5)	100 (4.1)	-0.6 (-1.9 to 0.6) [P=0.346]
Rheumatoid Arthritis				216 (15.3)	338 (14.0)	1.3 (-1.1 to 3.6) [P=0.288]
Ulcerative Colitis				105 (7.4)	130 (5.4)	2.0 (0.4 to 3.7) [P=0.012]

Abbreviations: CI, confidence interval; SD, standard deviation;

Samples includes all patients who received a biologic since quarter of first biosimilar launch in the drug class (Q3 2015 for filgrastim and Q4 2016 for infliximab).

[a] Patient received at least one biosimilar administration during sample period.

[b] Patient never received a biosimilar administration during sample period.

[c] Comparisons reflect two-group test of proportions. Mean-comparison (t) test used for previous use and risk score variables.

[d] Previous use reflects whether a patient who received at least one biosimilar administration also received a non-biosimilar product in the relevant drug class prior to their first biosimilar administration.

[e] Sample size except for risk score variable. See [f] below.

[f] Risk scores were based on the Department of Health and Human Services Hierarchical Conditions Categories (HHS-HCC) risk adjustment model using claims data from the preceding year.[14] Sample size for patients with a risk score was smaller as patients had to have one year of enrollment prior to first biologic administration (sample size by biosimilar administration for filgrastim was 23,081 and 45,180; for infliximab 1,527 and 12,894).

[g] Conditions are not mutually exclusive. Filgrastim related conditions are chemotherapy treatment with acute myeloid leukemia; bone marrow harvest; bone marrow transplant following chemotherapy in patients with nonmyeloid malignancies; congenital, cyclic, and idiopathic neutropenia; and chemotherapy treatment with nonmyeloid malignancy.

[h] Conditions are not mutually exclusive.

© 2021 Dean EB et al. *JAMA Network Open.*

eTable 2. Association Between Filgrastim Biosimilar Administration and Patient, Physician and Practice Characteristics With Patient Risk Score

	N	Adjusted Biosimilar rate (%)	Difference (pp)	95 CI		P-value
**Patient Characteristics**						
**Age**						
65-74 (ref)	122,527	30.5	0.0	-	-	-
75+	111,913	30.3	-0.2	-2.1	1.6	0.890
**Sex**						
Male (ref)	108,604	30.8	0.0	-	-	-
Female	125,836	30.1	-0.7	-2.5	1.2	0.783
**Race**						
White (ref)	206,720	30.7	0.0	-	-	-
Black	15,794	28.4	-2.3	-6.0	1.5	0.130
Other	11,926	29.2	-1.5	-5.5	2.5	0.439
**Dual Status**						
Non-Dual (ref)	214,420	30.2	0.0	-	-	-
Dual	20,020	32.6	2.4	-1.1	5.9	0.440
**Medical Condition**[a]						
Acute myeloid	7,052	35.6	5.3	-0.8	11.4	0.472
Neutropenia	100,875	29.1	-2.3	-4.2	-0.3	0.012
Nonmyeloid	171,003	31.1	2.5	-0.3	5.3	0.023
Transplantation	6,957	29.3	-1.2	-5.5	3.2	0.008
**Physician Characteristics**						
**Years Practicing**[b]						
Less than 15 years (ref)	28,144	31.8	0.0	-	-	-
Between 15 and 30 years	117,835	30.2	-1.6	-4.2	1.0	0.231
Greater than 30 years	88,461	30.3	-1.5	-4.3	1.3	0.110
**Sex**						
Male (ref)	172,568	30.5	0.0	-	-	-
Female	61,872	30.3	-0.1	-2.0	1.8	0.506
**Specialty**[c]						
Primary Care (ref)	39,314	32.9	0.0	-	-	-
Hematologist-Oncologist	120,894	29.6	-3.2	-5.8	-0.7	< 0.001
Oncologist	44,533	29.2	-3.7	-6.6	-0.7	0.002
Cancer-Treating Specialist	15,742	32.3	-0.5	-4.6	3.6	0.205
Other	13,957	32.2	-0.7	-4.4	3.0	0.711
**Hospital Ownership Status**						
Practices at independent practice (ref)	207,373	30.0	0.0	-	-	-
Practices at owned practice	27,067	33.5	3.4	0.6	6.3	0.126
**Physician Filgrastim Volume**[d]						
Low Volume (ref)	4,480	26.6	0.0	-	-	-
Medium Volume	86,960	30.2	3.6	1.5	5.6	< 0.001

© 2021 Dean EB et al. *JAMA Network Open.*

High Volume	143,000	30.7	4.1	1.8	6.3	< 0.001
**Practice Characteristics**						
**Practice Setting**						
Office (ref)	123,862	38.1	0.0	-	-	-
HOPD	110,578	21.8	-16.3	-18.4	-14.3	< 0.001
**Office Practice Characteristics**						
**Office Size**						
Less than 5 physicians(ref)	29,686	32.8	0.0	-	-	-
6-19 physicians	26,930	41.5	8.7	4.5	13.0	< 0.001
20-100 physicians	34,147	43.9	11.1	6.1	16.1	< 0.001
100+ physicians	33,099	35.7	2.9	-2.0	7.9	0.001
**Multispecialty Status**						
Single specialty (ref)	36,212	39.9	0.0	-	-	-
Multispecialty	87,650	37.9	-2.0	-5.9	2.0	0.400
**HOPD Practice Characteristics**[g]						
**Hospital Size**						
Less than 50 beds (ref)	10,897	15.2	0.0	-	-	-
51-100 beds	7,292	11.9	-3.3	-8.6	2.1	0.045
101-250 beds	26,900	21.7	6.5	2.3	10.8	0.007
Greater than 250 beds	65,489	23.3	8.2	3.8	12.5	0.007
**Hospital Ownership**						
Not-for-profit (ref)	86,261	21.7	0.0	-	-	-
For-profit	5,303	5.2	-16.5	-20.9	-12.1	< 0.001
Government	19,014	24.3	2.5	-1.1	6.1	0.924
**340B Status**[e]						
Non-340B Hospital (ref)	28,222	20.6	0.0	-	-	-
340B Hospital	82,356	21.6	1.0	-1.7	3.7	0.327
**AMC Status**[f]						
Non-AMC (ref)	78,987	21.7	0.0	-	-	-
AMC	31,591	20.6	-1.1	-4.4	2.1	0.678
**Hospital System Affiliation**						
Not affiliated (ref)	32,007	18.7	0.0	-	-	-
Affiliated	78,571	22.5	3.8	1.0	6.7	0.009

Results obtained from ordinary least-squares regressions of indicator of biosimilar administration on listed covariates as well as patient HCC risk score. Patient, physician, and practice setting results from regressions with those covariates. Office practice results from regression with office covariates and physician and practice covariates. HOPD results from regression with HOPD covariates and physician and practice covariates. All models also included year-quarter and state fixed effects and robust standard errors clustered at patient level.

Abbreviations: AMC, Academic Medical Center; CI, confidence interval; HOPD, hospital outpatient department; PP, percentage point; SD, standard deviation;

[a] Reference categories are patients without a condition. Filgrastim related conditions are not mutually exclusive and include chemotherapy treatment with acute myeloid leukemia; bone marrow transplant following chemotherapy in patients with nonmyeloid malignancies; congenital, cyclic, and idiopathic neutropenia; and

© 2021 Dean EB et al. *JAMA Network Open.*

chemotherapy treatment with nonmyeloid malignancy. Bone marrow harvest indication excluded due to small sample size.

[b] Years in practice indicates years since completion of medical school.

[c] Other cancer related specialties include gastroenterology, gynecology, proctology, urology, nephrology, endocrinology, and hematology.

[d] Low, medium and high volume defined as less than or equal to 1, between 1 and 5, and greater than 5 average monthly filgrastim administrations.

[e] 340B Status indicates that the hospital associated with the HOPD participated in the 340B Drug Pricing Program as identified through the Health Resources & Services Administration Office of Pharmacy Affairs.

[f] AMC indicates that the hospital associated with the HOPD is an Academic Medical Center.

eTable 3. Association Between Filgrastim Biosimilar Administration and Patient, Physician and Practice Characteristics, Model Specification Sensitivities

	(1)	(3)	(4)
	Original	Cluster at NPI	Cluster at facility
<u>**Patient characteristics**</u>			
**Age**			
65-74	0	0	0
	(.)	(.)	(.)
75+	-0.00296	-0.00296	-0.00296
	(0.00900)	(0.00824)	(0.00909)
**Gender**			
Male	0	0	0
	(.)	(.)	(.)
Female	-0.00499	-0.00499	-0.00499
	(0.00919)	(0.00856)	(0.00916)
**Race**			
White	0	0	0
	(.)	(.)	(.)
Black	-0.0222	-0.0222	-0.0222
	(0.0180)	(0.0185)	(0.0191)
Other	-0.0156	-0.0156	-0.0156
	(0.0203)	(0.0192)	(0.0225)
**Dual Status**			
Non-dual	0	0	0
	(.)	(.)	(.)
Dual	0.0245	0.0245	0.0245
	(0.0171)	(0.0155)	(0.0168)
**Nomyeloid**			
No nonmyloid	0	0	0
	(.)	(.)	(.)
Nonmyloid	0.0249	0.0249	0.0249
	(0.0138)	(0.0134)	(0.0170)
**Acute myeloid leukemia**			
No acute myloid leukemia	0	0	0
	(.)	(.)	(.)
Acute myeloid leukemia	0.0488	0.0488	0.0488
	(0.0292)	(0.0279)	(0.0299)
**Transplantation**			
No transplantation	0	0	0
	(.)	(.)	(.)
Transplantation	-0.00696	-0.00696	-0.00696
	(0.0212)	(0.0237)	(0.0351)
**Neutropenia**			
No Neutropenia	0	0	0
	(.)	(.)	(.)

© 2021 Dean EB et al. *JAMA Network Open.*

Neutropenia	-0.0203*	-0.0203*	-0.0203
	(0.00956)	(0.00892)	(0.0111)
**Physician characteristics**			
**Years practicing**			
Less than 15 years	0	0	0
	(.)	(.)	(.)
Between 15 and 30 years	-0.0180	-0.0180	-0.0180
	(0.0126)	(0.0170)	(0.0170)
Greater than 30 years	-0.0214	-0.0214	-0.0214
	(0.0134)	(0.0181)	(0.0190)
**Gender**			
Male	0	0	0
	(.)	(.)	(.)
Female	-0.00322	-0.00322	-0.00322
	(0.00937)	(0.0123)	(0.0117)
**Specialty**			
Primary care	0	0	0
	(.)	(.)	(.)
Hematologist-Oncologist	-0.0296*	-0.0296	-0.0296
	(0.0125)	(0.0152)	(0.0249)
Oncologist	-0.0338*	-0.0338	-0.0338
	(0.0145)	(0.0179)	(0.0306)
Cancer-treating specialist	-0.00313	-0.00313	-0.00313
	(0.0200)	(0.0248)	(0.0263)
Other	-0.00949	-0.00949	-0.00949
	(0.0178)	(0.0192)	(0.0207)
**Hospital Ownership Status**			
Practices at independent practice	0	0	0
	(.)	(.)	(.)
Practices at owned practice	0.0307*	0.0307	0.0307
	(0.0148)	(0.0195)	(0.0221)
**Physician Filgrastim Volume**			
Low volume	0	0	0
	(.)	(.)	(.)
Medium volume	0.0311**	0.0311**	0.0311*
	(0.00983)	(0.0107)	(0.0142)
High volume	0.0363***	0.0363**	0.0363*
	(0.0109)	(0.0121)	(0.0179)
**Practice characteristics**			
**Practice setting**			
Office	0	0	0

© 2021 Dean EB et al. *JAMA Network Open.*

	(.)	(.)	(.)
HOPD	-0.161***	-0.161***	-0.161***
	(0.0104)	(0.0134)	(0.0277)
<u>**Office practice characteristics**</u>			
**Office size**			
Less than 5 physicians	0	0	0
	(.)	(.)	(.)
6-19 physicians	0.0944***	0.0944***	0.0944
	(0.0209)	(0.0245)	(0.0496)
20-100 physicians	0.119***	0.119***	0.119*
	(0.0246)	(0.0300)	(0.0595)
100+ physicians	0.0400	0.0400	0.0400
	(0.0238)	(0.0281)	(0.0622)
**Multispecialty status**			
Single specialty	0	0	0
	(.)	(.)	(.)
Multispecialty	-0.0231	-0.0231	-0.0231
	(0.0192)	(0.0220)	(0.0448)
<u>**HOPD practice characteristics**</u>			
**Hospital size**			
Less than 50 beds	0	0	0
	(.)	(.)	(.)
51-100 beds	-0.0268	-0.0268	-0.0268
	(0.0269)	(0.0278)	(0.0412)
101-250 beds	0.0737***	0.0737**	0.0737*
	(0.0208)	(0.0234)	(0.0334)
Greater than 250 beds	0.0937***	0.0937***	0.0937**
	(0.0211)	(0.0232)	(0.0362)
**Hospital ownership**			
Not-for-profit	0	0	0
	(.)	(.)	(.)
For-profit	-0.174***	-0.174***	-0.174***
	(0.0213)	(0.0255)	(0.0366)
Government	0.0296	0.0296	0.0296
	(0.0174)	(0.0215)	(0.0392)
**340B status**			
Non-340B hospital	0	0	0
	(.)	(.)	(.)
340B hospital	0.00601	0.00601	0.00601
	(0.0133)	(0.0169)	(0.0308)
**AMC status**			

Non-AMC	0	0	0
	(.)	(.)	(.)
AMC	-0.0207	-0.0207	-0.0207
	(0.0159)	(0.0201)	(0.0368)
**Hospital system affiliation**			
Not affiliated	0	0	0
	(.)	(.)	(.)
Affiliated	0.0416**	0.0416*	0.0416
	(0.0142)	(0.0177)	(0.0311)
Observations, full sample	259173	259173	259173
Observations, office only sample	137375	137375	137375
Observations, HOPD only sample	121798	121798	121798

Standard errors in parentheses
* $p<0.05$, ** $p<0.01$, *** $p<0.001$

Results obtained from ordinary least-squares regressions of indicator of biosimilar administration on listed covariates. Patient, physician, and practice setting results from regression with patient and physician covariates and practice setting indicator. Office practice results from regression with office covariates and physician and practice covariates and includes only administrations in an office practice. HOPD results from regression with HOPD covariates and physician and practice covariates and includes only administrations in an HOPD. All models also included year-quarter and state fixed effects and robust standard errors. Column 1 clustered standard errors at the patient level, Columns 3 and 4 clustered standard errors at the physician and facility level.

Abbreviations: AMC, Academic Medical Center; HOPD, hospital outpatient department

© 2021 Dean EB et al. *JAMA Network Open.*

eTable 4. Association Between Filgrastim Biosimilar Administration and Patient, Physician and Practice Characteristics, Modifying Time Periods

	(1)	(2)	(3)	(4)	(5)
	Original	Remove 1st quarter	Only 2018	Only 2018 Q3-Q4	Only first 9 quarters
**Patient characteristics**					
**Age**					
65-74	0	0	0	0	0
	(.)	(.)	(.)	(.)	(.)
75+	-0.00296	-0.00335	-0.0109	-0.0152	0.000802
	(0.00900)	(0.00969)	(0.0167)	(0.0199)	(0.00919)
**Gender**					
Male	0	0	0	0	0
	(.)	(.)	(.)	(.)	(.)
Female	-0.00499	-0.00611	0.0138	0.0222	-0.0128
	(0.00919)	(0.00991)	(0.0171)	(0.0205)	(0.00930)
**Race**					
White	0	0	0	0	0
	(.)	(.)	(.)	(.)	(.)
Black	-0.0222	-0.0241	-0.0166	-0.0142	-0.0218
	(0.0180)	(0.0193)	(0.0335)	(0.0385)	(0.0178)
Other	-0.0156	-0.0150	0.00222	-0.0295	-0.0270
	(0.0203)	(0.0217)	(0.0402)	(0.0488)	(0.0189)
**Dual Status**					
Non-dual	0	0	0	0	0
	(.)	(.)	(.)	(.)	(.)
Dual	0.0245	0.0260	-0.00732	-0.0151	0.0392*
	(0.0171)	(0.0185)	(0.0327)	(0.0403)	(0.0160)
**Nomyeloid**					
No nonmyloid	0	0	0	0	0
	(.)	(.)	(.)	(.)	(.)
Nonmyloid	0.0249	0.0260	0.0251	0.0256	0.0216
	(0.0138)	(0.0148)	(0.0239)	(0.0268)	(0.0131)
**Acute myeloid leukemia**					
No acute myloid leukemia	0	0	0	0	0
	(.)	(.)	(.)	(.)	(.)
Acute myeloid leukemia	0.0488	0.0520	0.112*	0.0737	0.0284
	(0.0292)	(0.0319)	(0.0537)	(0.0565)	(0.0319)
**Transplantation**					
No transplantation	0	0	0	0	0
	(.)	(.)	(.)	(.)	(.)
Transplantation	-0.00696	-0.00431	-0.0159	0.0239	-0.0133

© 2021 Dean EB et al. *JAMA Network Open.*

	(0.0212)	(0.0224)	(0.0402)	(0.0550)	(0.0214)
**Neutropenia**					
No Neutropenia	0	0	0	0	0
	(.)	(.)	(.)	(.)	(.)
Neutropenia	-0.0203*	-0.0222*	-0.0311	-0.0375	-0.0140
	(0.00956)	(0.0103)	(0.0183)	(0.0217)	(0.00945)
**Physician characteristics**					
**Years practicing**	0	0	0	0	0
Less than 15 years	(.)	(.)	(.)	(.)	(.)
	-0.0180	-0.0196	-0.0280	-0.0371	-0.0170
Between 15 and 30 years	(0.0126)	(0.0133)	(0.0218)	(0.0263)	(0.0135)
	-0.0214	-0.0234	-0.0333	-0.0365	-0.0179
Greater than 30 years	(0.0134)	(0.0142)	(0.0239)	(0.0284)	(0.0141)
**Gender**					
Male	0	0	0	0	0
	(.)	(.)	(.)	(.)	(.)
Female	-0.00322	-0.00298	-0.00902	-0.0135	-0.00157
	(0.00937)	(0.0101)	(0.0179)	(0.0212)	(0.00960)
**Specialty**					
Primary care	0	0	0	0	0
	(.)	(.)	(.)	(.)	(.)
Hematologist-Oncologist	-0.0296*	-0.0322*	-0.0532*	-0.0664*	-0.0208
	(0.0125)	(0.0135)	(0.0222)	(0.0268)	(0.0133)
Oncologist	-0.0338*	-0.0387*	-0.0877**	-0.135***	-0.0155
	(0.0145)	(0.0157)	(0.0269)	(0.0324)	(0.0152)
Cancer-treating specialist	-0.00313	-0.00394	-0.0739*	-0.108*	0.0292
	(0.0200)	(0.0215)	(0.0371)	(0.0431)	(0.0206)
Other	-0.00949	-0.0105	-0.0472	-0.0349	0.00221
	(0.0178)	(0.0192)	(0.0326)	(0.0407)	(0.0196)
**Hospital Ownership Status**					
Practices at independent practice	0	0	0	0	0
	(.)	(.)	(.)	(.)	(.)
Practices at owned practice	0.0307*	0.0327*	0.0686*	0.0725*	0.0186
	(0.0148)	(0.0160)	(0.0273)	(0.0322)	(0.0170)
**Physician Filgrastim Volume**					
Low volume	0	0	0	0	0
	(.)	(.)	(.)	(.)	(.)
Medium volume	0.0311**	0.0343**	0.0245	0.0477	0.0304**
	(0.00983)	(0.0107)	(0.0206)	(0.0269)	(0.0102)
High volume	0.0363***	0.0394***	0.0376	0.0637*	0.0341**
	(0.0109)	(0.0119)	(0.0223)	(0.0287)	(0.0113)

© 2021 Dean EB et al. *JAMA Network Open.*

<u>**Practice characteristics**</u>					
**Practice setting**					
Office	0	0	0	0	0
	(.)	(.)	(.)	(.)	(.)
HOPD	-0.161***	-0.174***	-0.201***	-0.207***	-0.141***
	(0.0104)	(0.0111)	(0.0198)	(0.0238)	(0.0106)
<u>**Office practice characteristics**</u>					
**Office size**					
Less than 5 physicians	0	0	0	0	0
	(.)	(.)	(.)	(.)	(.)
6-19 physicians	0.0944***	0.102***	0.0923*	0.0554	0.101***
	(0.0209)	(0.0227)	(0.0418)	(0.0468)	(0.0210)
20-100 physicians	0.119***	0.133***	0.0891	0.0614	0.127***
	(0.0246)	(0.0267)	(0.0467)	(0.0508)	(0.0247)
100+ physicians	0.0400	0.0455	0.119*	0.107*	-0.00364
	(0.0238)	(0.0257)	(0.0464)	(0.0538)	(0.0242)
**Multispecialty status**					
Single specialty	0	0	0	0	0
	(.)	(.)	(.)	(.)	(.)
Multispecialty	-0.0231	-0.0283	-0.0305	-0.0544	-0.0156
	(0.0192)	(0.0208)	(0.0383)	(0.0429)	(0.0190)
<u>**HOPD practice characteristics**</u>					
**Hospital size**					
Less than 50 beds	0	0	0	0	0
	(.)	(.)	(.)	(.)	(.)
51-100 beds	-0.0268	-0.0277	-0.110*	-0.0738	0.00185
	(0.0269)	(0.0287)	(0.0545)	(0.0641)	(0.0239)
101-250 beds	0.0737***	0.0777***	0.0481	0.0775	0.0689***
	(0.0208)	(0.0223)	(0.0471)	(0.0561)	(0.0177)
Greater than 250 beds	0.0937***	0.0994***	0.0563	0.0991	0.110***
	(0.0211)	(0.0227)	(0.0475)	(0.0555)	(0.0178)
**Hospital ownership**					
Not-for-profit	0	0	0	0	0
	(.)	(.)	(.)	(.)	(.)
For-profit	-0.174***	-0.189***	-0.362***	-0.358***	-0.0998***
	(0.0213)	(0.0227)	(0.0343)	(0.0441)	(0.0210)
Government	0.0296	0.0310	0.0439	0.0527	0.0324
	(0.0174)	(0.0186)	(0.0332)	(0.0392)	(0.0167)
**340B status**					
Non-340B hospital	0	0	0	0	0
	(.)	(.)	(.)	(.)	(.)

© 2021 Dean EB et al. *JAMA Network Open.*

340B hospital	0.00601	0.00597	-0.0399	-0.0734*	0.0202
	(0.0133)	(0.0143)	(0.0268)	(0.0332)	(0.0124)
**AMC status**					
Non-AMC	0	0	0	0	0
	(.)	(.)	(.)	(.)	(.)
AMC	-0.0207	-0.0249	-0.0436	-0.0424	-0.0152
	(0.0159)	(0.0173)	(0.0298)	(0.0356)	(0.0150)
**Hospital system affiliation**					
Not affiliated	0	0	0	0	0
	(.)	(.)	(.)	(.)	(.)
Affiliated	0.0416**	0.0445**	0.0678*	0.0823*	0.0265*
	(0.0142)	(0.0152)	(0.0283)	(0.0344)	(0.0130)
Observations, full sample	259173	239127	70111	34359	171626
Observations, office only sample	137375	126495	36070	17458	92224
Observations, HOPD only sample	121798	112632	34041	16901	79402

Standard errors in parentheses
* p<0.05, ** p<0.01, *** p<0.001

Results obtained from ordinary least-squares regressions of indicator of biosimilar administration on listed covariates. Patient, physician, and practice setting results from regression with patient and physician covariates and practice setting indicator. Office practice results from regression with office covariates and physician and practice covariates and includes only administrations in an office practice. HOPD results from regression with HOPD covariates and physician and practice covariates and includes only administrations in an HOPD. All models also included year-quarter and state fixed effects and robust standard errors clustered at the patient level.

Abbreviations: AMC, Academic Medical Center; HOPD, hospital outpatient department

© 2021 Dean EB et al. *JAMA Network Open.*

eTable 5. Association Between Filgrastim Biosimilar Administration and Patient, Physician and Practice Characteristics, Inclusion/Exclusion Based on Volume

	(1)	(2)	(3)	(4)
	Original	Remove Low vol bene's	Remove Low vol physicians	Only high vol physicians
**Patient characteristics**				
**Age**				
65-74	0	0	0	0
	(.)	(.)	(.)	(.)
75+	-0.00296	-0.00259	-0.00352	-0.00706
	(0.00900)	(0.00941)	(0.00940)	(0.0127)
**Gender**				
Male	0	0	0	0
	(.)	(.)	(.)	(.)
Female	-0.00499	-0.00537	-0.00545	-0.00000549
	(0.00919)	(0.00962)	(0.00961)	(0.0128)
**Race**				
White	0	0	0	0
	(.)	(.)	(.)	(.)
Black	-0.0222	-0.0240	-0.0236	-0.0278
	(0.0180)	(0.0188)	(0.0189)	(0.0266)
Other	-0.0156	-0.0167	-0.0162	-0.0178
	(0.0203)	(0.0212)	(0.0212)	(0.0288)
**Dual Status**				
Non-dual	0	0	0	0
	(.)	(.)	(.)	(.)
Dual	0.0245	0.0277	0.0246	0.0182
	(0.0171)	(0.0180)	(0.0179)	(0.0231)
**Nonmyeloid malignancy**				
No nonmyloid malignancy	0	0	0	0
	(.)	(.)	(.)	(.)
Nonmyloid malignancy	0.0249	0.0252	0.0247	0.0215
	(0.0138)	(0.0143)	(0.0143)	(0.0194)
**Acute myeloid leukemia**				
No acute myloid leukemia	0	0	0	0
	(.)	(.)	(.)	(.)
Acute myeloid leukemia	0.0488	0.0485	0.0449	0.0570
	(0.0292)	(0.0301)	(0.0303)	(0.0392)
**Transplantation**				
No transplanation	0	0	0	0
	(.)	(.)	(.)	(.)

© 2021 Dean EB et al. *JAMA Network Open.*

Transplantation	-0.00696	-0.00540	-0.00828	-0.0254
	(0.0212)	(0.0217)	(0.0219)	(0.0274)
**Neutropenia**				
No Neutropenia	0	0	0	0
	(.)	(.)	(.)	(.)
Neutropenia	-0.0203*	-0.0211*	-0.0208*	-0.0136
	(0.00956)	(0.00998)	(0.00999)	(0.0133)
**Physician characteristics**				
**Years practicing**				
Less than 15 years	0	0	0	0
	(.)	(.)	(.)	(.)
Between 15 and 30 years	-0.0180	-0.0190	-0.0192	-0.0144
	(0.0126)	(0.0133)	(0.0136)	(0.0196)
Greater than 30 years	-0.0214	-0.0220	-0.0218	-0.0266
	(0.0134)	(0.0141)	(0.0144)	(0.0206)
**Gender**				
Male	0	0	0	0
	(.)	(.)	(.)	(.)
Female	-0.00322	-0.00369	-0.00383	-0.0134
	(0.00937)	(0.00980)	(0.00987)	(0.0138)
**Specialty**				
Primary care	0	0	0	0
	(.)	(.)	(.)	(.)
Hematologist-Oncologist	-0.0296*	-0.0302*	-0.0346*	-0.0373
	(0.0125)	(0.0130)	(0.0135)	(0.0195)
Oncologist	-0.0338*	-0.0349*	-0.0393*	-0.0558*
	(0.0145)	(0.0152)	(0.0156)	(0.0221)
Cancer-treating specialist	-0.00313	-0.00427	-0.00944	-0.00391
	(0.0200)	(0.0208)	(0.0217)	(0.0307)
Other	-0.00949	-0.00955	-0.00900	-0.0308
	(0.0178)	(0.0191)	(0.0212)	(0.0324)
**Hospital Ownership Status**				
Practices at independent practice	0	0	0	0
	(.)	(.)	(.)	(.)
Practices at owned practice	0.0307*	0.0325*	0.0294	0.0474*
	(0.0148)	(0.0157)	(0.0158)	(0.0213)
**Physician Filgrastim Volume**				
Low volume	0	0	0	
	(.)	(.)	(.)	
Medium volume	0.0311**	0.0158	0.0295	

© 2021 Dean EB et al. *JAMA Network Open.*

	(0.00983)	(0.0136)	(0.0467)	
High volume	0.0363***	0.0198	0.0333	0
	(0.0109)	(0.0144)	(0.0468)	(.)
**Practice characteristics**				
**Practice setting**				
Office	0	0	0	0
	(.)	(.)	(.)	(.)
HOPD	-0.161***	-0.162***	-0.162***	-0.185***
	(0.0104)	(0.0109)	(0.0108)	(0.0142)
**Office practice characteristics**				
**Office size**				
Less than 5 physicians	0	0	0	0
	(.)	(.)	(.)	(.)
6-19 physicians	0.0944***	0.0945***	0.0946***	0.0842**
	(0.0209)	(0.0218)	(0.0221)	(0.0315)
20-100 physicians	0.119***	0.117***	0.119***	0.0985**
	(0.0246)	(0.0256)	(0.0261)	(0.0375)
100+ physicians	0.0400	0.0376	0.0405	0.0164
	(0.0238)	(0.0249)	(0.0252)	(0.0356)
**Multispecialty status**				
Single specialty	0	0	0	0
	(.)	(.)	(.)	(.)
Multispecialty	-0.0231	-0.0221	-0.0254	-0.0458
	(0.0192)	(0.0200)	(0.0204)	(0.0293)
**HOPD practice characteristics**				
**Hospital size**				
Less than 50 beds	0	0	0	0
	(.)	(.)	(.)	(.)
51-100 beds	-0.0268	-0.0281	-0.0273	-0.0747*
	(0.0269)	(0.0281)	(0.0281)	(0.0316)
101-250 beds	0.0737***	0.0765***	0.0759***	0.0570*
	(0.0208)	(0.0218)	(0.0218)	(0.0289)
Greater than 250 beds	0.0937***	0.0959***	0.0928***	0.0848**
	(0.0211)	(0.0221)	(0.0221)	(0.0293)
**Hospital ownership**				
Not-for-profit	0	0	0	0
	(.)	(.)	(.)	(.)
For-profit	-0.174***	-0.178***	-0.180***	-0.163***
	(0.0213)	(0.0227)	(0.0225)	(0.0299)
Government	0.0296	0.0291	0.0290	0.0228
	(0.0174)	(0.0182)	(0.0182)	(0.0230)

© 2021 Dean EB et al. *JAMA Network Open.*

**340B status**				
Non-340B hospital	0	0	0	0
	(.)	(.)	(.)	(.)
340B hospital	0.00601	0.00375	0.00656	-0.0102
	(0.0133)	(0.0141)	(0.0140)	(0.0185)
**AMC status**				
Non-AMC	0	0	0	0
	(.)	(.)	(.)	(.)
AMC	-0.0207	-0.0196	-0.0185	-0.0278
	(0.0159)	(0.0168)	(0.0167)	(0.0217)
**Hospital system affiliation**				
Not affiliated	0	0	0	0
	(.)	(.)	(.)	(.)
Affiliated	0.0416**	0.0407**	0.0422**	0.0236
	(0.0142)	(0.0149)	(0.0148)	(0.0188)
Observations, full sample	259173	247574	246032	157884
Observations, office only sample	137375	131920	130374	77515
Observations, HOPD only sample	121798	115654	115658	80369

Standard errors in parentheses
* p<0.05, ** p<0.01, *** p<0.001

Results obtained from ordinary least-squares regressions of indicator of biosimilar administration on listed covariates. Patient, physician, and practice setting results from regression with patient and physician covariates and practice setting indicator. Office practice results from regression with office covariates and physician and practice covariates and includes only administrations in an office practice. HOPD results from regression with HOPD covariates and physician and practice covariates and includes only administrations in an HOPD. All models also included year-quarter and state fixed effects and robust standard errors clustered at the patient level. For columns 2 and 3, the beneficiaries or physicians with the lowest volume (representing 5% of the sample) was dropped. For Column 4, only physicians in the High Filgrastim Volume category were included.

Abbreviations: AMC, Academic Medical Center; HOPD, hospital outpatient department

© 2021 Dean EB et al. *JAMA Network Open.*

eTable 6. Association Between Infliximab Biosimilar Administration and Patient, Physician and Practice Characteristics With Patient Risk Score

	N	Adjusted Biosimilar rate (%)	Difference (pp)	95 CI		P-value
**Patient Characteristics**						
**Age**						
65-74 (ref)	90,865	5.0	0.0	-	-	-
75+	68,821	5.1	0.1	-0.6	0.7	0.861
**Sex**						
Male (ref)	50,026	4.7	0.0	-	-	-
Female	109,660	5.2	0.5	-0.2	1.2	0.150
**Race**						
White (ref)	145,846	5.1	0.0	-	-	-
Black	7,790	4.9	-0.2	-1.6	1.3	0.830
Other	6,050	4.0	-1.1	-2.8	0.6	0.195
**Dual Status**[a]						
Non-Dual (ref)	151,143	5.1	0.0	-	-	-
Dual	8,543	4.3	-0.8	-2.2	0.6	0.250
**Medical Condition**[a]						
Ankylosing Spondylitis	6,947	4.2	-0.9	-2.4	0.7	0.260
Crohn's Disease	27,225	3.5	-1.8	-2.9	-0.7	0.001
Psoriatic Arthritis	22,223	5.6	0.6	-0.4	1.7	0.233
Rheumatoid Arthritis	106,406	5.1	0.1	-0.9	1.2	0.826
Ulcerative colitis	17,753	4.1	-1.0	-2.2	0.2	0.093
**Physician Characteristics**						
**Years Practicing**[b]						
Less than 15 years (ref)	21,965	4.0	0.0	-	-	-
Between 15 and 30 years	65,867	5.5	1.5	0.6	2.5	0.001
Greater than 30 years	71,854	4.9	0.9	0.0	1.9	0.054
**Sex**						
Male (ref)	114,279	5.6	0.0	-	-	-
Female	45,407	3.7	-1.9	-2.6	-1.2	< 0.001
**Specialty**						
Primary Care (ref)	37,439	5.1	0.0	-	-	-
Dermatologist	1,366	3.3	-1.7	-5.1	1.6	0.314
Gastroenterologist	21,763	4.3	-0.8	-2.0	0.4	0.201
Rheumatologist	87,201	4.9	-0.2	-1.4	1.1	0.785
Other	11,917	8.0	2.9	1.3	4.5	< 0.001
**Hospital Ownership Status**						

Practices at independent practice (ref)	75,473	5.3	0.0	-	-	-
Practices at owned practice	84,213	4.8	-0.5	-1.6	0.5	0.294
**Physician Infliximab Volume**[c]						
Low Volume (ref)	33,202	4.5	0.0	-	-	-
Medium Volume	42,282	4.6	0.1	-0.7	1.0	0.769
High Volume	84,202	5.5	1.1	0.1	2.0	0.023
**Practice Characteristics**						
**Practice Setting**						
Office (ref)	100,774	4.0	0.0	-	-	-
HOPD	58,912	6.9	2.9	2.1	3.6	< 0.001
**Office Practice Characteristics**						
**Office Size**						
Less than 5 physicians (ref)	46,247	4.2	0.0	-	-	-
6-19 physicians	22,081	2.5	-1.7	-2.6	-0.7	0.001
20-100 physicians	16,274	6.6	2.4	0.8	4.0	0.003
100+ physicians	16,172	3.2	-1.0	-2.5	0.5	0.192
**Multispecialty Status**						
Single specialty (ref)	61,826	4.6	0.0	-	-	-
Multispecialty	38,948	3.2	-1.4	-2.6	-0.2	0.023
**HOPD Practice Characteristics**						
**Hospital Size**						
Less than 50 beds (ref)	5,808	3.6	0.0	-	-	-
51-100 beds	5,535	4.9	1.2	-1.3	3.8	0.339
101-250 beds	15,282	8.5	4.9	2.8	7.0	< 0.001
Greater than 250 beds	32,287	6.7	3.1	1.1	5.0	0.002
**Hospital Ownership**						
Not-for-profit (ref)	47,877	6.1	0.0	-	-	-
For-profit	3,242	16.3	10.2	6.0	14.4	< 0.001
Government	7,793	6.3	0.2	-1.5	1.8	0.838
**340B Status**[d]						
Non-340B Hospital (ref)	16,148	10.6	0.0	-	-	-
340B Hospital	42,764	5.2	-5.3	-7.0	-3.6	< 0.001
**AMC Status**[e]						
Non-AMC (ref)	45,076	7.8	0.0	-	-	-
AMC	13,836	3.2	-4.6	-5.8	-3.4	< 0.001
**Hospital System Affiliation**						
Not affiliated (ref)	14,961	7.0	0.0	-	-	-
Affiliated	48,006	6.6	-0.4	-1.9	1.1	0.586

Results obtained from ordinary least-squares regressions of indicator of biosimilar administration on listed covariates as well as patient HCC risk score. Patient, physician, and practice setting

© 2021 Dean EB et al. *JAMA Network Open.*

results from regressions with those covariates. Office practice results from regression with office covariates and physician and practice covariates. HOPD results from regression with HOPD covariates and physician and practice covariates. All models also included year-quarter and state fixed effects and robust standard errors clustered at the patient level.

Abbreviations: AMC, Academic Medical Center; CI, confidence interval; HOPD, hospital outpatient department; PP, percentage point; SD, standard deviation;

[a] Reference categories are patients without a condition. Infliximab related conditions are not mutually exclusive.

[b] Years in practice indicates years since completion of medical school.

[c] Low, medium and high volume defined as less than or equal to 1, between 1 and 2, and greater than 2 average monthly infliximab administrations.

[d] 340B Status indicates that the hospital associated with the HOPD participated in the 340B Drug Pricing Program as identified through the Health Resources & Services Administration Office of Pharmacy Affairs.

[e] AMC indicates that the hospital associated with the HOPD is an Academic Medical Center.

© 2021 Dean EB et al. *JAMA Network Open.*

eTable 7. Association Between Infliximab Biosimilar Administration and Patient, Physician and Practice Characteristics, Model Specification Sensitivities

**Association between infliximab biosimilar administration and patient, physician and practice characteristics, model specification sensitivities**			
	(1)	(2)	(3)
	Original	Cluster at NPI	Cluster at setting
**Patient characteristics**			
**Age**			
65-74	0	0	0
	(.)	(.)	(.)
75+	0.00216	0.00216	0.00216
	(0.00310)	(0.00310)	(0.00330)
**Gender**			
Male	0	0	0
	(.)	(.)	(.)
Female	0.00437	0.00437	0.00437
	(0.00340)	(0.00333)	(0.00359)
**Race**			
White	0	0	0
	(.)	(.)	(.)
Black	-0.000518	-0.000518	-0.000518
	(0.00719)	(0.00682)	(0.00869)
Other	-0.0126	-0.0126	-0.0126
	(0.00854)	(0.00841)	(0.00811)
**Dual Status**			
Non-dual	0	0	0
	(.)	(.)	(.)
Dual	-0.00535	-0.00535	-0.00535
	(0.00695)	(0.00649)	(0.00686)
**Ulcerative Colitis**			
No ulcerative colitis	0	0	0
	(.)	(.)	(.)
Ulcerative colitis	-0.0101	-0.0101	-0.0101
	(0.00592)	(0.00615)	(0.00654)
**Rheumatoid arthritis**			
No rheumatoid arthritis	0	0	0
	(.)	(.)	(.)
Rheumatoid arthritis	0.00254	0.00254	0.00254
	(0.00524)	(0.00535)	(0.00608)

© 2021 Dean EB et al. *JAMA Network Open.*

**Ankylosing spondylitis**			
No ankylosing spondylitis	0	0	0
	(.)	(.)	(.)
Ankylosing spondylitis	-0.00705	-0.00705	-0.00705
	(0.00750)	(0.00742)	(0.00809)
**Psoriatic arthritis**			
No psoriatic arthritis	0	0	0
	(.)	(.)	(.)
Psoriatic arthritis	0.00830	0.00830	0.00830
	(0.00530)	(0.00517)	(0.00531)
**Physician characteristics**			
**Years practicing**			
Less than 15 years	0	0	0
	(.)	(.)	(.)
Between 15 and 30 years	0.0150***	0.0150*	0.0150*
	(0.00448)	(0.00615)	(0.00626)
Greater than 30 years	0.00895	0.00895	0.00895
	(0.00463)	(0.00627)	(0.00593)
**Gender**			
Male	0	0	0
	(.)	(.)	(.)
Female	-0.0182***	-0.0182***	-0.0182**
	(0.00342)	(0.00543)	(0.00579)
**Specialty**			
Primary care	0	0	0
	(.)	(.)	(.)
Hematologist-Oncologist	-0.00378	-0.00378	-0.00378
	(0.0188)	(0.0197)	(0.0202)
Oncologist	-0.00839	-0.00839	-0.00839
	(0.00594)	(0.00673)	(0.00716)
Cancer-treating specialist	-0.00464	-0.00464	-0.00464
	(0.00616)	(0.0105)	(0.00991)
Other	0.0294***	0.0294**	0.0294*
	(0.00818)	(0.00941)	(0.0121)
**Hospital Ownership Status**			
Practices at independent practice	0	0	0
	(.)	(.)	(.)
Practices at owned practice	-0.00483	-0.00483	-0.00483
	(0.00503)	(0.00888)	(0.00833)
**Physician Filgrastim Volume**			
Low volume	0	0	0

© 2021 Dean EB et al. *JAMA Network Open.*

	(.)	(.)	(.)
Medium volume	0.00323	0.00323	0.00323
	(0.00407)	(0.00443)	(0.00485)
High volume	0.0125**	0.0125*	0.0125
	(0.00463)	(0.00603)	(0.00715)
**Practice characteristics**			
**Practice setting**			
Office	0	0	0
	(.)	(.)	(.)
HOPD	0.0295***	0.0295***	0.0295***
	(0.00375)	(0.00593)	(0.00848)
**Office practice characteristics**			
**Office size**			
Less than 5 physicians	0	0	0
	(.)	(.)	(.)
6-19 physicians	-0.0181***	-0.0181**	-0.0181
	(0.00487)	(0.00673)	(0.0109)
20-100 physicians	0.0258**	0.0258*	0.0258
	(0.00807)	(0.0115)	(0.0298)
100+ physicians	-0.0101	-0.0101	-0.0101
	(0.00733)	(0.00965)	(0.0206)
**Multispecialty status**			
Single specialty	0	0	0
	(.)	(.)	(.)
Multispecialty	-0.0150*	-0.0150	-0.0150
	(0.00606)	(0.00801)	(0.0195)
**HOPD practice characteristics**			
**Hospital size**			
Less than 50 beds	0	0	0
	(.)	(.)	(.)
51-100 beds	0.0132	0.0132	0.0132
	(0.0123)	(0.0159)	(0.0198)
101-250 beds	0.0517***	0.0517***	0.0517**
	(0.0103)	(0.0126)	(0.0163)
Greater than 250 beds	0.0335***	0.0335**	0.0335*
	(0.00914)	(0.0114)	(0.0138)
**Hospital ownership**			
Not-for-profit	0	0	0
	(.)	(.)	(.)
For-profit	0.108***	0.108***	0.108***
	(0.0209)	(0.0250)	(0.0325)

© 2021 Dean EB et al. *JAMA Network Open.*

Government	-0.00182	-0.00182	-0.00182
	(0.00790)	(0.0134)	(0.0180)
**340B status**			
Non-340B hospital	0	0	0
	(.)	(.)	(.)
340B hospital	-0.0542***	-0.0542***	-0.0542**
	(0.00833)	(0.0130)	(0.0175)
**AMC status**			
Non-AMC	0	0	0
	(.)	(.)	(.)
AMC	-0.0474***	-0.0474***	-0.0474***
	(0.00585)	(0.00764)	(0.0108)
**Hospital system affiliation**			
Not affiliated	0	0	0
	(.)	(.)	(.)
Affiliated	-0.00416	-0.00416	-0.00416
	(0.00731)	(0.0108)	(0.0140)
Observations, full sample	174973	174973	174973
Observations, office only sample	110586	110586	110586
Observations, HOPD only sample	64387	64387	64387

Standard errors in parentheses
* p<0.05, ** p<0.01, *** p<0.001

Results obtained from ordinary least-squares regressions of indicator of biosimilar administration on listed covariates. Patient, physician, and practice setting results from regression with patient and physician covariates and practice setting indicator. Office practice results from regression with office covariates and physician and practice covariates and includes only administrations in an office practice. HOPD results from regression with HOPD covariates and physician and practice covariates and includes only administrations in an HOPD. All models also included year-quarter and state fixed effects and robust standard errors. Column 1 clustered standard errors at the patient level, Columns 3 and 4 clustered standard errors at the physician and facility level.

Abbreviations: AMC, Academic Medical Center; HOPD, hospital outpatient department

© 2021 Dean EB et al. *JAMA Network Open.*

eTable 8. Association Between Infliximab Biosimilar Administration and Patient, Physician and Practice Characteristics, Modifying Time Periods

	(1)	(2)	(3)	(4)
	Original	Remove 1st quarter	Only 2018	Only 2018 Q3-Q4
**Patient characteristics**				
**Age**				
65-74	0	0	0	0
	(.)	(.)	(.)	(.)
75+	0.00216	0.00240	0.00430	0.00564
	(0.00310)	(0.00343)	(0.00501)	(0.00591)
**Gender**				
Male	0	0	0	0
	(.)	(.)	(.)	(.)
Female	0.00437	0.00497	0.00584	0.00132
	(0.00340)	(0.00378)	(0.00556)	(0.00655)
**Race**				
White	0	0	0	0
	(.)	(.)	(.)	(.)
Black	-0.000518	-0.000605	0.00339	-0.00130
	(0.00719)	(0.00798)	(0.0119)	(0.0137)
Other	-0.0126	-0.0136	-0.0179	-0.0235
	(0.00854)	(0.00942)	(0.0126)	(0.0140)
**Dual Status**				
Non-dual	0	0	0	0
	(.)	(.)	(.)	(.)
Dual	-0.00535	-0.00578	-0.0127	-0.00650
	(0.00695)	(0.00774)	(0.0112)	(0.0140)
**Ulcerative Colitis**				
No ulcerative colitis	0	0	0	0
	(.)	(.)	(.)	(.)
Ulcerative colitis	-0.0101	-0.0112	-0.0171	-0.0257*
	(0.00592)	(0.00652)	(0.00955)	(0.0113)
**Rheumatoid arthritis**				
No rheumatoid arthritis	0	0	0	0
	(.)	(.)	(.)	(.)
Rheumatoid arthritis	0.00254	0.00268	0.00196	0.000287
	(0.00524)	(0.00579)	(0.00855)	(0.00991)
**Ankylosing spondylitis**				
No ankylosing spondylitis	0	0	0	0
	(.)	(.)	(.)	(.)

© 2021 Dean EB et al. *JAMA Network Open.*

Ankylosing spondylitis	-0.00705	-0.00781	-0.00683	-0.0112
	(0.00750)	(0.00826)	(0.0122)	(0.0133)
**Psoriatic arthritis**				
No psoriatic arthritis	0	0	0	0
	(.)	(.)	(.)	(.)
Psoriatic arthritis	0.00830	0.00909	0.0142	0.0144
	(0.00530)	(0.00588)	(0.00885)	(0.0104)
**Physician characteristics**				
**Years practicing**				
Less than 15 years	0	0	0	0
	(.)	(.)	(.)	(.)
Between 15 and 30 years	0.0150***	0.0165***	0.0192**	0.0190*
	(0.00448)	(0.00492)	(0.00689)	(0.00839)
Greater than 30 years	0.00895	0.00969	0.00736	0.00234
	(0.00463)	(0.00509)	(0.00718)	(0.00878)
**Gender**				
Male	0	0	0	0
	(.)	(.)	(.)	(.)
Female	-0.0182***	-0.0200***	-0.0264***	-0.0274***
	(0.00342)	(0.00379)	(0.00554)	(0.00660)
**Specialty**				
Primary care	0	0	0	0
	(.)	(.)	(.)	(.)
Hematologist-Oncologist	-0.00378	-0.00381	0.00778	0.0189
	(0.0188)	(0.0208)	(0.0317)	(0.0392)
Oncologist	-0.00839	-0.00902	-0.00824	-0.00366
	(0.00594)	(0.00655)	(0.00906)	(0.0111)
Cancer-treating specialist	-0.00464	-0.00471	-0.00217	-0.00130
	(0.00616)	(0.00690)	(0.0102)	(0.0126)
Other	0.0294***	0.0326***	0.0427***	0.0415**
	(0.00818)	(0.00903)	(0.0122)	(0.0141)
**Hospital Ownership Status**				
Practices at independent practice	0	0	0	0
	(.)	(.)	(.)	(.)
Practices at owned practice	-0.00483	-0.00569	-0.00313	0.00289
	(0.00503)	(0.00565)	(0.00853)	(0.0104)
**Physician Filgrastim Volume**				
Low volume	0	0	0	0
	(.)	(.)	(.)	(.)
Medium volume	0.00323	0.00386	0.00174	0.000687
	(0.00407)	(0.00453)	(0.00666)	(0.00792)

High volume	0.0125**	0.0140**	0.0132	0.00585
	(0.00463)	(0.00514)	(0.00739)	(0.00878)
**<u>Practice characteristics</u>**				
**Practice setting**				
Office	0	0	0	0
	(.)	(.)	(.)	(.)
HOPD	0.0295***	0.0325***	0.0455***	0.0606***
	(0.00375)	(0.00416)	(0.00590)	(0.00699)
**<u>Office practice characteristics</u>**				
**Office size**	0	0	0	0
Less than 5 physicians	(.)	(.)	(.)	(.)
	-0.0181***	-0.0202***	-0.0236**	-0.0173
6-19 physicians	(0.00487)	(0.00540)	(0.00807)	(0.00922)
	0.0258**	0.0288**	0.0554***	0.0744***
20-100 physicians	(0.00807)	(0.00892)	(0.0127)	(0.0141)
	-0.0101	-0.0111	-0.00470	0.0112
100+ physicians	(0.00733)	(0.00814)	(0.0112)	(0.0126)
**Multispecialty status**	0	0	0	0
Single specialty	(.)	(.)	(.)	(.)
	-0.0150*	-0.0169*	-0.0283**	-0.0394***
Multispecialty	(0.00606)	(0.00671)	(0.00912)	(0.0100)
**<u>HOPD practice characteristics</u>**				
**Hospital size**				
Less than 50 beds	0	0	0	0
	(.)	(.)	(.)	(.)
51-100 beds	0.0132	0.0122	0.00117	-0.00694
	(0.0123)	(0.0137)	(0.0193)	(0.0231)
101-250 beds	0.0517***	0.0559***	0.0637***	0.0625**
	(0.0103)	(0.0114)	(0.0162)	(0.0196)
Greater than 250 beds	0.0335***	0.0357***	0.0540***	0.0598**
	(0.00914)	(0.0101)	(0.0147)	(0.0182)
**Hospital ownership**				
Not-for-profit	0	0	0	0
	(.)	(.)	(.)	(.)
For-profit	0.108***	0.123***	0.191***	0.251***
	(0.0209)	(0.0236)	(0.0345)	(0.0396)
Government	-0.00182	-0.00231	-0.0127	-0.0175
	(0.00790)	(0.00878)	(0.0126)	(0.0159)
**340B status**				

© 2021 Dean EB et al. *JAMA Network Open.*

Non-340B hospital	0	0	0	0
	(.)	(.)	(.)	(.)
340B hospital	-0.0542***	-0.0604***	-0.0801***	-0.0873***
	(0.00833)	(0.00928)	(0.0130)	(0.0157)
**AMC status**				
Non-AMC	0	0	0	0
	(.)	(.)	(.)	(.)
AMC	-0.0474***	-0.0525***	-0.0781***	-0.0922***
	(0.00585)	(0.00647)	(0.0104)	(0.0132)
**Hospital system affiliation**				
Not affiliated	0	0	0	0
	(.)	(.)	(.)	(.)
Affiliated	-0.00416	-0.00486	-0.00291	-0.000661
	(0.00731)	(0.00810)	(0.0115)	(0.0140)
Observations, full sample	174973	157577	79119	38813
Observations, office only sample	110586	99471	49499	24142
Observations, HOPD only sample	64387	58106	29620	14671

Standard errors in parentheses
* p<0.05, ** p<0.01, *** p<0.001

Results obtained from ordinary least-squares regressions of indicator of biosimilar administration on listed covariates. Patient, physician, and practice setting results from regression with patient and physician covariates and practice setting indicator. Office practice results from regression with office covariates and physician and practice covariates and includes only administrations in an office practice. HOPD results from regression with HOPD covariates and physician and practice covariates and includes only administrations in an HOPD. All models also included year-quarter and state fixed effects and robust standard errors clustered at the patient level.

Abbreviations: AMC, Academic Medical Center; HOPD, hospital outpatient department

© 2021 Dean EB et al. *JAMA Network Open.*

eTable 9. Association Between Infliximab Biosimilar Administration and Patient, Physician and Practice Characteristics, Inclusion/Exclusion Based on Volume

	(1)	(2)	(3)	(4)
	Original	Remove Low vol bene's	Remove Low vol physicians	Only high vol physicians
**Patient characteristics**				
**Age**				
65-74	0	0	0	0
	(.)	(.)	(.)	(.)
75+	0.00216	0.00154	0.00168	-0.0000446
	(0.00310)	(0.00323)	(0.00318)	(0.00445)
**Gender**				
Male	0	0	0	0
	(.)	(.)	(.)	(.)
Female	0.00437	0.00417	0.00527	0.00451
	(0.00340)	(0.00356)	(0.00348)	(0.00497)
**Race**				
White	0	0	0	0
	(.)	(.)	(.)	(.)
Black	-0.000518	0.000264	-0.000680	-0.00639
	(0.00719)	(0.00758)	(0.00736)	(0.00969)
Other	-0.0126	-0.0133	-0.0134	-0.0294**
	(0.00854)	(0.00899)	(0.00881)	(0.0105)
**Dual Status**				
Non-dual	0	0	0	0
	(.)	(.)	(.)	(.)
Dual	-0.00535	-0.00557	-0.00627	0.00739
	(0.00695)	(0.00741)	(0.00721)	(0.0121)
**Ulcerative Colitis**				
No ulcerative colitis	0	0	0	0
	(.)	(.)	(.)	(.)
Ulcerative colitis	-0.0101	-0.0104	-0.00936	-0.00398
	(0.00592)	(0.00623)	(0.00614)	(0.0111)
**Rheumatoid arthritis**				
No rheumatoid arthritis	0	0	0	0
	(.)	(.)	(.)	(.)
Rheumatoid arthritis	0.00254	0.00305	0.00190	-0.00178
	(0.00524)	(0.00551)	(0.00541)	(0.00789)
**Ankylosing spondylitis**				
No ankylosing spondylitis	0	0	0	0
	(.)	(.)	(.)	(.)

© 2021 Dean EB et al. *JAMA Network Open.*

Ankylosing spondylitis	-0.00705	-0.00859	-0.00625	-0.0143
	(0.00750)	(0.00782)	(0.00770)	(0.0102)
**Psoriatic arthritis**				
No psoriatic arthritis	0	0	0	0
	(.)	(.)	(.)	(.)
Psoriatic arthritis	0.00830	0.00809	0.00757	0.00392
	(0.00530)	(0.00554)	(0.00536)	(0.00725)
**Physician characteristics**				
**Years practicing**				
Less than 15 years	0	0	0	0
	(.)	(.)	(.)	(.)
Between 15 and 30 years	0.0150***	0.0138**	0.0152**	0.0224***
	(0.00448)	(0.00472)	(0.00472)	(0.00675)
Greater than 30 years	0.00895	0.00899	0.00995*	0.0147*
	(0.00463)	(0.00487)	(0.00486)	(0.00704)
**Gender**				
Male	0	0	0	0
	(.)	(.)	(.)	(.)
Female	-0.0182***	-0.0177***	-0.0186***	-0.0289***
	(0.00342)	(0.00358)	(0.00356)	(0.00506)
**Specialty**				
Primary care	0	0	0	0
	(.)	(.)	(.)	(.)
Hematologist-Oncologist	-0.00378	-0.00187	-0.000479	-0.0295
	(0.0188)	(0.0197)	(0.0199)	(0.0248)
Oncologist	-0.00839	-0.00918	-0.00954	-0.0158
	(0.00594)	(0.00618)	(0.00626)	(0.0142)
Cancer-treating specialist	-0.00464	-0.00427	-0.00487	-0.00572
	(0.00616)	(0.00646)	(0.00649)	(0.0110)
Other	0.0294***	0.0267**	0.0291**	0.0276
	(0.00818)	(0.00870)	(0.00887)	(0.0166)
**Hospital Ownership Status**				
Practices at independent practice	0	0	0	0
	(.)	(.)	(.)	(.)
Practices at owned practice	-0.00483	-0.00568	-0.00401	-0.0122
	(0.00503)	(0.00528)	(0.00525)	(0.00699)
**Physician Filgrastim Volume**				
Low volume	0	0	0	
	(.)	(.)	(.)	
Medium volume	0.00323	0.00320	0.00356	

© 2021 Dean EB et al. *JAMA Network Open.*

	(0.00407)	(0.00425)	(0.00439)	
High volume	0.0125**	0.0141**	0.0137**	0
	(0.00463)	(0.00488)	(0.00497)	(.)
**Practice characteristics**				
**Practice setting**				
Office	0	0	0	0
	(.)	(.)	(.)	(.)
HOPD	0.0295***	0.0295***	0.0300***	0.0261***
	(0.00375)	(0.00393)	(0.00387)	(0.00565)
**Office practice characteristics**				
**Office size**				
Less than 5 physicians	0	0	0	0
	(.)	(.)	(.)	(.)
6-19 physicians	-0.0181***	-0.0189***	-0.0191***	-0.0137
	(0.00487)	(0.00510)	(0.00502)	(0.00762)
20-100 physicians	0.0258**	0.0242**	0.0249**	0.0384**
	(0.00807)	(0.00848)	(0.00832)	(0.0139)
100+ physicians	-0.0101	-0.0120	-0.0105	0.0107
	(0.00733)	(0.00767)	(0.00760)	(0.0129)
**Multispecialty status**				
Single specialty	0	0	0	0
	(.)	(.)	(.)	(.)
Multispecialty	-0.0150*	-0.0133*	-0.0148*	-0.0310**
	(0.00606)	(0.00635)	(0.00625)	(0.0103)
**HOPD practice characteristics**				
**Hospital size**				
Less than 50 beds	0	0	0	0
	(.)	(.)	(.)	(.)
51-100 beds	0.0132	0.0118	0.0146	0.0164
	(0.0123)	(0.0129)	(0.0128)	(0.0214)
101-250 beds	0.0517***	0.0512***	0.0545***	0.0511**
	(0.0103)	(0.0108)	(0.0107)	(0.0165)
Greater than 250 beds	0.0335***	0.0337***	0.0344***	0.0462**
	(0.00914)	(0.00959)	(0.00945)	(0.0152)
**Hospital ownership**				
Not-for-profit	0	0	0	0
	(.)	(.)	(.)	(.)
For-profit	0.108***	0.108***	0.104***	0.0638
	(0.0209)	(0.0220)	(0.0217)	(0.0339)
Government	-0.00182	-0.000982	-0.00288	-0.000464
	(0.00790)	(0.00832)	(0.00823)	(0.0138)

© 2021 Dean EB et al. *JAMA Network Open.*

**340B status**				
Non-340B hospital	0	0	0	0
	(.)	(.)	(.)	(.)
340B hospital	-0.0542***	-0.0535***	-0.0540***	-0.0841***
	(0.00833)	(0.00876)	(0.00858)	(0.0137)
**AMC status**				
Non-AMC	0	0	0	0
	(.)	(.)	(.)	(.)
AMC	-0.0474***	-0.0470***	-0.0484***	-0.0591***
	(0.00585)	(0.00610)	(0.00590)	(0.0105)
**Hospital system affiliation**				
Not affiliated	0	0	0	0
	(.)	(.)	(.)	(.)
Affiliated	-0.00416	-0.00420	-0.00384	-0.0341**
	(0.00731)	(0.00768)	(0.00762)	(0.0122)
Observations, full sample	174973	164993	167221	92142
Observations, office only sample	110586	104663	106090	62901
Observations, HOPD only sample	64387	60330	61131	29241

Standard errors in parentheses
* p<0.05, ** p<0.01, *** p<0.001

Results obtained from ordinary least-squares regressions of indicator of biosimilar administration on listed covariates. Patient, physician, and practice setting results from regression with patient and physician covariates and practice setting indicator. Office practice results from regression with office covariates and physician and practice covariates and includes only administrations in an office practice. HOPD results from regression with HOPD covariates and physician and practice covariates and includes only administrations in an HOPD. All models also included year-quarter and state fixed effects and robust standard errors clustered at the patient level. For columns 2 and 3, the beneficiaries or physicians with the lowest volume (representing 5% of the sample) was dropped. For Column 4, only physicians in the High Filgrastim Volume category were included.

Abbreviations: AMC, Academic Medical Center; HOPD, hospital outpatient department

© 2021 Dean EB et al. *JAMA Network Open.*
